# Novel delafossite structured visible-light sensitive AgFeO_2_/PPAC nanocomposite for efficient adsorptive photocatalytic degradation of cationic and anionic dyes

**DOI:** 10.1038/s41598-026-60824-z

**Published:** 2026-07-09

**Authors:** Nora A. El-Mahdy, El-Sayed R. El-Gharkawy, Magda A. Akl

**Affiliations:** Chemistry Department, Faculty of Science, University of Mansoura, Mansoura, 35516 Egypt

**Keywords:** Pomegranate peel activated carbon, Delafossite AgFeO_2_, Neutral Red, Brilliant Blue R, Photocatalytic degradation, Wastewater, Chemistry, Environmental sciences, Materials science, Nanoscience and technology

## Abstract

In this work, a novel delafossite AgFeO_2_-based pomegranate peel activated carbon nanocomposite (AgFeO_2_/PPAC) was synthesized via a hydrothermal route. Structural and surface characterization (XRD, SEM–EDX, BET, TGA, and FTIR) of PPAC and AgFeO_2_/PPAC nanocomposite indicate successful deposition of AgFeO_2_ onto a porous carbon matrix with high surface area. The AgFeO_2_/PPAC nanocomposite was evaluated for the removal of the anionic Brilliant Blue R (BBR) and the cationic Neutral Red (NR) dyes from aqueous solutions under visible light. The AgFeO_2_/PPAC nanocomposite shows high removal efficiency toward both dyes in single and binary systems. The experimental data fit well with the Langmuir isotherm model, suggesting monolayer adsorption under the studied conditions. The maximum adsorption capacities are 798.6 mg g^−1^ for NR and 598 mg g^−1^ for BBR. Kinetic analysis shows good agreement with the pseudo-second-order model (PSO), indicating that surface interactions contribute to the adsorption process. Thermodynamic parameters indicate a spontaneous and endothermic process. The overall removal performance is attributed to the combined contribution of adsorption and photocatalytic activity. The AgFeO_2_/PPAC nanocomposite demonstrates stable performance over repeated cycles, indicating good reusability. These findings suggest that the developed material is a promising candidate for wastewater treatment applications**.**

**Supplementary Information:**

The online version contains supplementary material available at 10.1038/s41598-026-60824-z.

## Introduction

The growing discharge of untreated industrial and urban waste into freshwater systems continues to position water pollution among the most pressing global environmental crises^1,2^. This contamination poses a direct threat to public health, disturbs ecological balance, and constrains the availability of potable water, particularly in areas with underdeveloped infrastructure^2^. Among various pollution sources, effluents generated by textile, leather, and dye-manufacturing industries are of particular concern due to their high content of synthetic organic dyes. These compounds possess complex aromatic structures, exhibit strong chemical stability, and resist natural biodegradation, leading to their long-term persistence in aquatic environments^3^. Synthetic dyes represent a significant subset of these contaminants their presence impedes light transmission in water bodies, suppresses photosynthetic activity, and can yield toxic by-products that endanger both aquatic organisms and human populations^4^. The extensive application of dyes in industries such as textiles, paper, plastics, and food processing means that even minimal concentrations can cause significant visual pollution and disrupt fundamental aquatic processes^5,6^. Two dyes of specific interest are Neutral Red (NR) and Brilliant Blue R (BBR). Neutral Red, a cationic phenazine dye employed in microbiological staining, demonstrates considerable environmental persistence and is associated with potential cytotoxic and mutagenic impacts^7,8^. Conversely, BBR, an anionic triphenylmethane dye used in textiles and bio-assays, is recalcitrant to standard degradation methods and may exhibit genotoxicity upon accumulation in aquatic environments^9,10^. The effective removal of these dyes from wastewater is therefore an urgent environmental priority. A wide range of physicochemical and biological techniques has been developed for dye removal, including coagulation–flocculation^11^, chemical oxidation^12^, membrane filtration^13^, and biological treatment^14^, adsorption^15^, and photocatalytic degradation^16,17^. Among these approaches, photocatalytic degradation has emerged as a highly promising technique due to its ability to degradation of organic pollutants through reactive species generated under light irradiation^18,19^. Photocatalysis relies on semiconductor materials that absorb light and generate electron–hole pairs, which subsequently produce reactive oxygen species such as hydroxyl radicals and superoxide anions. These species are reported to contribute to dye molecules degradation into harmless end products such as carbon dioxide and water under light irradiation^20^.

Conventional photocatalysts, including TiO_2_ and ZnO, have demonstrated effective dye degradation performance^21,22^. However, their practical application is limited by insufficient visible-light absorption and rapid recombination of photo generated charge carriers. To overcome these drawbacks, recent researches have focused on developing modified photocatalysts through coupling with carbon-based materials or constructing heterostructured metal oxides^23,24^. Carbon supports not only enhance adsorption capacity but also improve charge separation efficiency by acting as electron acceptors, thereby promoting photocatalytic degradation under visible-light irradiation^25^.

In this context, activated carbon derived from agricultural biomass has attracted considerable attention as a sustainable and low-cost support material. Pomegranate peel represents an abundant agro-waste precursor with high carbon content, natural phenolic compounds, and diverse surface functional groups^26^. Activated carbon produced from pomegranate peel typically exhibits high surface area, well-developed porosity, and strong affinity toward both cationic and anionic dyes. These characteristics facilitate efficient pollutant adsorption and enhance mass transfer during photocatalytic reactions. Furthermore, biomass-derived activated carbon contributes to waste valorization and aligns with circular economy principles^27^. Activated carbons derived from such biomass typically exhibit high surface area, tunable porosity, and a strong affinity for various dyes and heavy metals^28^. Supporting this, the work by Akl et al., has demonstrated that activated carbons and modified organoclay from agro-residues can achieve high removal efficiencies in dye-laden systems^29,30^. Recent advances have demonstrated that integrating photocatalytically active metal oxides with biomass-derived activated carbon can significantly improve overall degradation efficiency.

The delafossite-type oxides, generally expressed by the formula ABO_2_ (A = Cu or Ag; B = Fe, Al, or Cr), possess a layered crystal structure composed of linearly coordinated A-site cations and edge-sharing BO_6_ octahedral^31^. This unique arrangement endows delafossite materials with favorable electronic properties, including narrow band gaps and efficient charge transport under visible-light irradiation^32^. Among various metal oxides, delafossite AgFeO_2_ has emerged as a promising visible-light-responsive photocatalyst due to its narrow band gap, layered crystal structure, and favorable charge transport properties. The incorporation of Fe^3^⁺ ions within the delafossite lattice also can facilitie magnetic separation, as suggested in previous studies^33^. Combining delafossite AgFeO_2_ with activated carbon therefore offers a synergistic platform that unites strong adsorption, efficient photocatalytic degradation, and practical reusability**.** Furthermore, post-synthesis modifications including chemical activation, impregnation with metals, or magnetization can significantly enhance adsorbent capacity. Also, they introduce functionality for magnetic separation, and expand their overall utility^34,35^. Recently, a novel delafossite-AgFeO_2_ decorated cellulose acetate ultrafiltration membrane has been successfully used for efficient water purification. In this study, the overall enhancement of cellulose acetate (CA) UF performance has been explored (i.e., permeation, antifouling, antibacterial, and dye rejection properties) through incorporation of AgFeO_2_ nanoparticles (NPs) in the membrane matrix^36^.

Despite extensive research conducted on carbon-supported photocatalysts, the majority of reported systems exhibit limitations in terms of visible-light efficiency, inadequate integration of adsorption and photocatalysis, and a paucity of performance evaluation in binary dye systems. Furthermore, the combination of delafossite AgFeO_2_ with biomass-derived activated carbon has received comparatively little exploration, particularly with regard to the simultaneous removal of cationic and anionic dyes. The novelty of this work lies in the in-situ hydrothermal integration of delafossite AgFeO_2_ with biomass-derived activated carbon, enabling improved interfacial contact and combined adsorption–photocatalytic performance under visible light. The developed system was evaluated for the removal of NR and BBR dyes under visible light conditions under different operational parameters. The structural, surface, and thermal properties of the nanocomposite were characterized using standard techniques. Kinetic, isotherm, and thermodynamic analyses were conducted to elucidate the governing degradation mechanisms. Besides, the reusability and performance of the material were evaluated under repeated cycles and simulated water systems.

Accordingly, the main objectives were: (i) Preparation of a delafossite AgFeO_2_ pomegranate peel activated carbon (AgFeO_2_/PPAC) nanocomposite; (ii) Characterization of the synthesized AgFeO_2_/PPAC nanocomposite using FT-IR, XRD, SEM–EDX, BET, and TGA; (iii) Investigation of the effects of pH, AgFeO_2_/PPAC dose, initial dye concentration, contact time, and temperature on the adsorptive photocatalytic degradation of NR and BBR dyes; (iv) Statistical analysis of isotherm and kinetic models using χ^2^, SSE, MSE, and Hybrid error functions; (v) Evaluation of the material’s stability and performance across repeated cycles; (vi) Examination of the adsorptive photocatalytic activity of AgFeO_2_/PPAC in single and binary dye systems under visible light; (vii) Elucidation of the plausible mechanism of the adsorption and photocatalytic degration of NR and BBR using AgFeO_2_/PPAC and (viii) Assessment of AgFeO_2_/PPAC nanocomposite performance in real water matrices.

## Experimental

### Materials

Pomegranate peels were obtained from local vendors in Egypt. All reagents used in the nanocomposite synthesis were analytical grade and used as received. AgNO_3_ (99.8%) and FeCl_3_·6H_2_O (99%) were obtained from Merck Group, while H_3_PO_4_ (85%), HCl (37%), NaOH (98%), KCl (99.5%), along with Brilliant Blue R (C_45_H_44_N_3_NaO_7_S_2_) and Neutral Red (C_15_H_17_ClN_4_·HCl), were purchased from Sigma-Aldrich. Glycerol was supplied by Advent International. Double distilled water was used as the non-solvent during preparation. The classifications, chemical structures and absorption wavelengths of the used organic dyes are displayed in Table S1.

### Synthesis of delafossite AgFeO_2_-grafted pomegranate peel activated carbon (AgFeO_2_/PPAC) nanocomposite

#### Preparation of pomegranate peel activated carbon (PPAC)

**Fig. 1:**
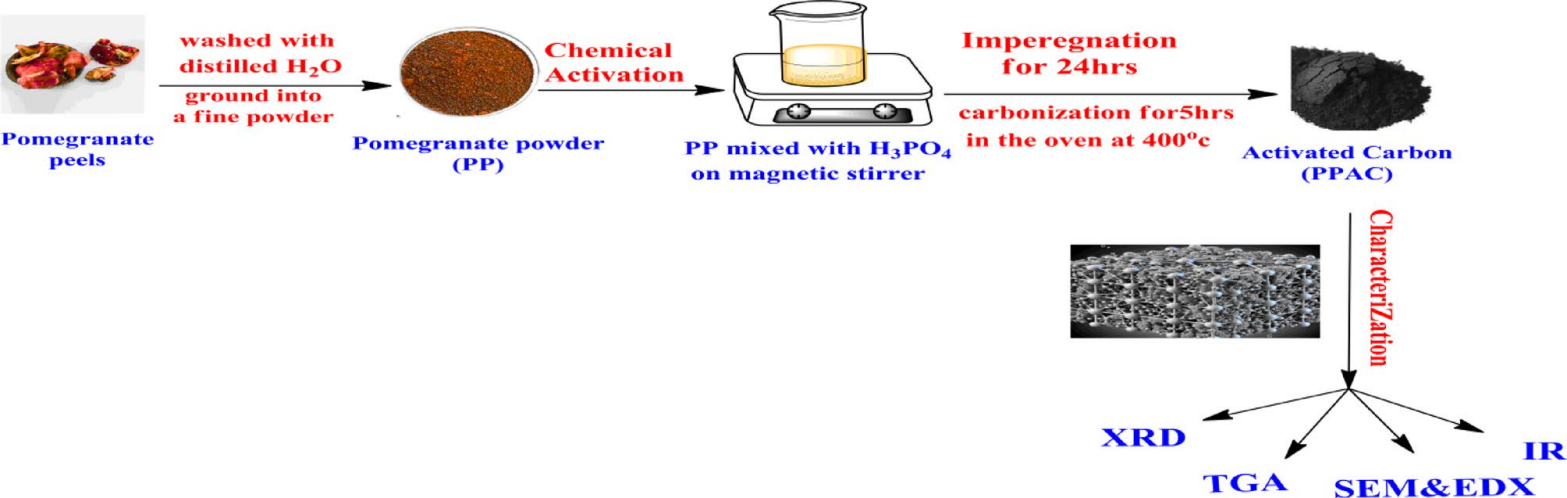
Synthesis of pomegranate peel activated carbon (PPAC).

As shown in Fig. 1, pomegranate peels were thoroughly washed, dried at 50 °C, and ground into a fine powder. To prepare the activated carbon (PPAC), the powder was impregnated with 80% phosphoric acid (H_3_PO_4_) at a 1:2 weight ratio for 24 h. The mixture was then carbonized at 400 °C for 5 h. After cooling, the resulting carbon was washed with distilled water until a neutral pH was reached and finally dried at 110 °C until it reached a constant weight to yield PPAC^37^.

#### Hydrothermal synthesis of AgFeO_2_/PPAC nanocomposite

**Fig. 2:**
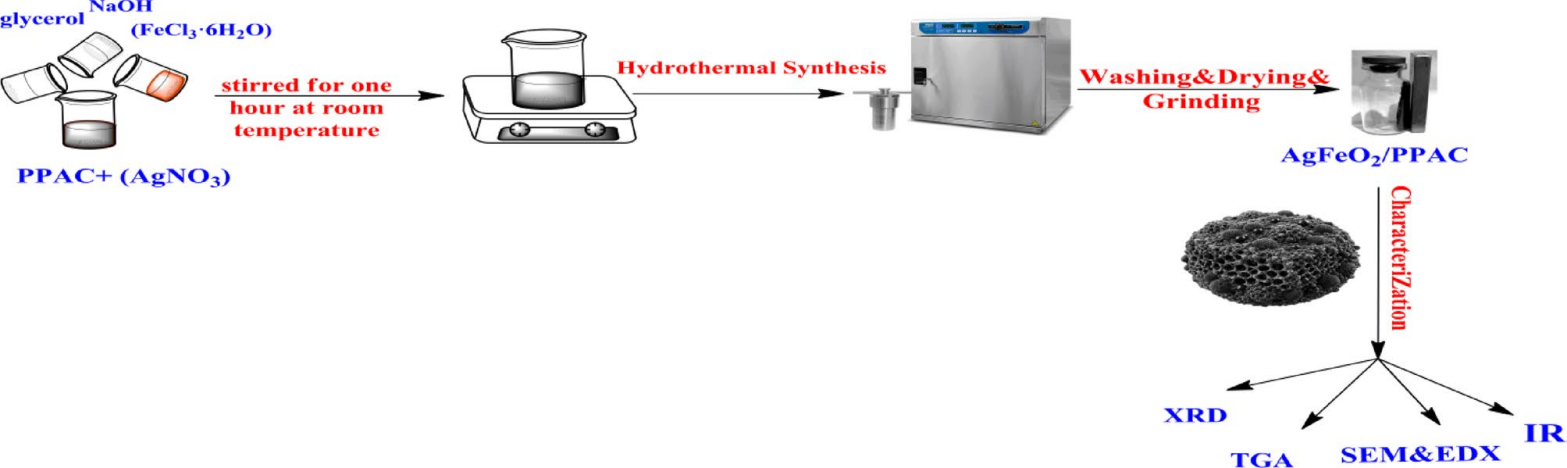
Hydrothermal synthesis of delafossite AgFeO_2_/PPAC nanocomposite.

For synthesis of the AgFeO_2_/PPAC nanocomposite , 5 mmol each of AgNO_3_ and FeCl_3_·6H_2_O were dissolved in deionized water. NaOH (10 g/40 mL) was added dropwise under stirring, followed by 12 mL of glycerol. PPAC was added, and the slurry was stirred for 1 h before being transferred to a Teflon-lined autoclave and heated at 180 °C for 24 h. The product was filtered, washed, and dried at 150 °C (Fig. 2)^38,39^.

### Material characterization

The specific surface area of the samples was determined using BET analysis with a Quantachrome–NOVA 2000 Series analyzer. Surface morphology was examined by SEM (Quanta FEG-250). FTIR spectra of powdered samples were recorded using a Spectrum One FTIR spectrometer from PerkinElmer. Thermal stability of PPAC and AgFeO_2_/PPAC was evaluated by TGA (PerkinElmer TGA 4000) over 30–800 °C. Crystalline structure was analyzed by XRD using a Bruker Corporation D2 PHASER diffractometer with Cu Kα radiation (λ = 1.54060). Elemental distribution of PPAC and AgFeO_2_/PPAC was assessed by EDX (IT100LA) coupled with SEM at an accelerating voltage of 20 kV. UV–Vis absorption spectra of NR, BBR, and their mixture were measured in the 190–900 nm range using a PerkinElmer 550 spectrophotometer. A neodymium N52 permanent magnet with dimensions of 50 × 20 × 7 mm and a pulling force of 15 kg was used for the external magnetic separation and recovery of the AgFeO_2_/PPAC nanocomposite after the combined sorption and photocatalytic degradation of the dyes. The point of zero charge pH_PZC_ of the delafossite AgFeO_2_/PPAC nanocomposite was evaluated as previously reported^40,41^.

#### Adsorptive-photocatalytic degradation procedures

To evaluate the contribution of adsorption and photocatalytic degradation during dye removal, batch experiments were conducted using Neutral Red (NR) and Brilliant Blue R (BBR) as model pollutants. The adsorption performance of raw pomegranate peel powder (Raw-PP), activated carbon (PPAC), and the AgFeO_2_/PPAC nanocomposite was first examined under dark conditions. In each experiment, 0.01 g of adsorbent was added to 10 mL dye solution (100 µg /mL) at the optimized pH values for each dye. The suspensions were shaken at 150 rpm and 30 °C for 24 h to ensure adsorption equilibrium. After separation by filtration or magnetic recovery, the residual dye concentration was measured using UV–Vis spectrophotometry, and the adsorption capacity was calculated according to Eq. (3).

The photocatalytic degradation performance of AgFeO_2_/PPAC nanocomposite was subsequently investigated under visible-light irradiation using a 150 W Xenon lamp (400–700 nm, 100 mW cm^−2^) positioned 10 cm above the reaction system. For each experiment, 0.005 g of photocatalyst was dispersed in 10 mL dye solution inside transparent stoppered bottles. The lamp was switched on to initiate the photocatalytic process, and samples were collected at regular intervals after magnetic separation of the catalyst. Control experiments were also carried out under identical irradiation conditions without photocatalyst. The degradation efficiency (D_e_ %) and the rate constant (k) were determined using Eqs. (1) and (2)^42^:

1 $$\% D_{e} = \left( {1 - \frac{C}{{C_{0} }}} \right) \times 100$$

2 $${ - }{\mathrm{ln}}\left( {{\mathrm{C/C}}_{{\mathrm{0}}} } \right){\text{ = Kt}}$$

where C_0_ and C represent the dye concentration at initial (0) and time (t), respectively.

The photocatalytic activity of AgFeO_2_/PPAC nanocomposite was further evaluated using a binary dye system containing 400 µg/ml NR and 300 µg/ml BBR under the optimized experimental conditions. The pH of the mixed solution was adjusted using diluted HCl or NaOH(0.1 M)^15^ before irradiation, and the residual dye concentration was monitored by UV–Vis spectroscopy. The magnetic behaviour of the AgFeO_2_/PPAC nanocomposite enabled rapid separation and efficient recovery of the photocatalyst after each treatment cycle^16^.

### Optimization of photocatalytic degradation parameters and equilibrium studies

The photocatalytic efficiency of the AgFeo_2_/PPAC nanocomposite was systematically evaluated by examining key operational parameters. The effects of pH (2–11), dosage (0.0025, 0.005, 0.0075, and 0.01 g), concentration (150–500 µg/mL), and temperature (25–65 °C) were evaluated. All experiments were conducted in batch mode using 10 mL dye solutions under visible light irradiation, with residual dye concentrations determined spectrophotometrically. The removal capacity at equilibrium, q_e_ (mg/g), was calculated using Eq. 3:

3 $${q}_{e}= ({C}_{o}-{C}_{e}) \times \frac{V}{m}$$

where C_0_ and C_e_ represent the initial and equilibrium dye concentrations (µg/ml), V is the solution volume (L), and m is the mass of photocatalyst (g)^43^.

### Isotherm, kinetic, and thermodynamic studies

The experimental data were further analyzed using various isotherm Langmuir (Eq. 4), Freundlich (Eq. 5), Temkin (Eq. 6), and Dubinin–Radushkevich (D–R) (Eqs. 7–9).The value of R_L_ serves as a key indicator of process favorability, where 0 < R_L_ < 1 signifies favorable adsorption as in Eq. (10). The kinetic models were pseudo-first-order (Eq. 11), pseudo-second-order (Eq. 12), Intra particle Diffusion (IPD) (Eq. 13), and Elovich (Eq. 14) to understand the surface interactions.

4 $$\frac{{{\mathrm{C}}_{{\mathrm{e}}} }}{{{\mathrm{q}}_{{\mathrm{e}}} }}{ = }\frac{{1}}{{{\mathrm{K}}_{{\mathrm{l}}} {\mathrm{q}}_{{\mathrm{m}}} }}{ + }\frac{{{\mathrm{C}}_{{\mathrm{e}}} }}{{{\mathrm{q}}_{{\mathrm{m}}} }}$$

5 $${\mathrm{lnq}}_{{\mathrm{e}}} {\text{ = lnK}}_{{\mathrm{f}}} { + }\frac{{{\mathrm{lnC}}_{{\mathrm{e}}} }}{{\mathrm{n}}}$$

6 $${\mathrm{q}}_{{\mathrm{e}}} {\text{ = B lnK}}_{{\mathrm{T}}} {\text{ + B ln C}}_{{\mathrm{e}}}$$

7 $$\ln q_{e} = \ln q_{m} - K_{DR} \varepsilon^{2}$$

8 $$\varepsilon = RT\ln \left( {1 + \frac{1}{{C_{e} }}} \right)$$

9 $${\text{E = }}\frac{{1}}{{\sqrt {{\mathrm{2K}}_{{{\mathrm{DR}}}} } }}$$

10 $$R_{L} = \frac{1}{{1 + K_{L} \times c_{e} }}$$

11 $$\frac{{1}}{{{\mathrm{q}}_{{\mathrm{t}}} }}{ = }\frac{{{\mathrm{K}}_{{1}} }}{{{\mathrm{q}}_{{\mathrm{e}}} {\mathrm{t}}}}{ + }\frac{{1}}{{{\mathrm{q}}_{{\mathrm{e}}} }}$$

12 $$\frac{{\mathrm{t}}}{{{\mathrm{q}}_{{\mathrm{e}}} }}{ = }\frac{{1}}{{{\mathrm{K}}_{{2}} {\mathrm{q}}_{{\mathrm{e}}}^{{2}} }}{ + }\frac{{\mathrm{t}}}{{{\mathrm{q}}_{{\mathrm{e}}} }}$$

13 $${\mathrm{q}}_{{\mathrm{t}}} {\text{ = K}}_{{{\mathrm{id}}}} {\mathrm{t}}^{{{0}{\mathrm{.5}}}} {\text{ + C}}$$

14 $$q_{t} = \frac{1}{\beta }\ln \left( {\alpha \beta } \right) + \frac{1}{\beta }\ln t$$

For the Langmuir model, q_m_ represents the maximum adsorption capacity (mg/g), K_L_ is the Langmuir constant related to adsorption affinity (L/mg), and R_L_ is the dimensionless separation factor indicating the favorability of the adsorption process. In the Freundlich model, K_F_ and n (or 1/n) are the Freundlich constants associated with removal capacity and intensity, respectively, Regarding the Temkin model, the parameter B is defined as RT/b_T_, where K_T_ is the Temkin equilibrium binding constant (L/g), b_T_ is the constant related to the heat of adsorption (J/mol), R is the universal gas constant (8.314 J/mol.K), and T is the absolute temperature (K). For the Dubinin–Radushkevich (D–R) model, K_DR_ is a constant related to the mean adsorption energy (mol^2^/J^2^), ε is the Polanyi potential, and E is the mean free energy of adsorption (J/mol)^44^. In the pseudo-first-order and pseudo-second-order models, q_t_ denotes the removal capacity (mg/g) at time t, while K_1_ and K_2_ represent their respective rate constants. For the intra-particle diffusion model, k_id_ is the intra-particle diffusion rate constant (mg/g·min^0.5^) and C is a constant related to the boundary layer thickness (mg/g). In the Elovich model, α corresponds to the initial adsorption rate (mg/g·min) and β is the desorption constant related to the extent of surface coverage (g/mg)^45^.

### Thermodynamic studies

Gibbs free energy (ΔG°), enthalpy (ΔH°), and entropy (ΔS°) were derived from the van’t Hoff plots using Eqs. 15 and 16:

15 $$\Delta {\mathrm{G}}^{{\mathrm{o}}} = - {\mathrm{RT}}\;{\mathrm{ln}}\;{\mathrm{K}}_{{\mathrm{c}}}$$

16 $${\text{ ln}}\;{\mathrm{K}}_{{\mathrm{c}}} = \frac{{\Delta S^{{\mathrm{o}}} }}{{\mathrm{R}}} - \frac{{\Delta H^{{\mathrm{o}}} }}{{{\mathrm{RT}}}}$$

where R is the universal gas constant (8.314 J/mol·K), T is the absolute temperature (K), and Kc is the equilibrium constant. The values of ΔH° and ΔS° were determined from the slope and intercept of the van’t Hoff plot (lnK_c_ versus 1/T)^46^.

### Statistical validation

To confirm the reliability of the models, the goodness-of-fit was evaluated using four statistical error functions *viz*. sum of squares error (SSE), mean square error (MSE), hybrid fractional error (HYBRID), and chi-square statistic (χ^2^) (Eqs. 17–20):

17 $${\text{SSE = }}\sum\limits_{{{\text{i = 1}}}}^{{\mathrm{n}}} {{\mathrm{(q}}_{{{\mathrm{e,exp}}}} } - {\mathrm{q}}_{{{\mathrm{e,calc}}}} )^{2}$$

18 $${\text{MSE = }}\frac{1}{{\mathrm{n}}}\sum\limits_{{{\text{i = 1}}}}^{{\mathrm{n}}} {{\mathrm{(q}}_{{{\mathrm{e,exp}}}} } - {\mathrm{q}}_{{{\mathrm{e,calc}}}} )^{2}$$

19 $${\text{ x}}^{2} = \sum\limits_{{{\text{i = 1}}}}^{{\mathrm{n}}} {\frac{{{\mathrm{(q}}_{{{\mathrm{e,exp}}}} - {\mathrm{q}}_{{{\mathrm{e,calc}}}} )^{2} }}{{{\mathrm{q}}_{{{\mathrm{e,cal}}}} }}}$$

20 $${\text{HYBRID = }}\frac{{100}}{{{\mathrm{N}} - {\mathrm{P}}}}\sum\limits_{{{\text{i = 1}}}}^{{\mathrm{n}}} {\left[ {\frac{{{\mathrm{(q}}_{{{\mathrm{e,exp}}}} - {\mathrm{q}}_{{{\mathrm{e,calc}}}} )^{2} }}{{{\mathrm{q}}_{{{\mathrm{e,cal}}}} }}} \right]}$$

In these Equations, qe_exp_ represents the experimentally observed equilibrium adsorption capacity (mg g^−1^), qe_calc_ is the equilibrium adsorption capacity predicted by the model (mg g^−1^), n is the total number of experimental observations, and p is the number of parameters in the fitted model^47^.

### Reusability and stability

Reusability was assessed over five cycles, with the catalyst regenerated using ethanol, 0.1 M NaOH, or 0.1 M HCl.

### Application in real water matrices

For practical validation, the AgFeO_2_/PPAC nanocomposite’s performance was tested in spiked tap water, seawater, and industrial wastewater matrices. Real-water samples (tap, sea, and industrial) were pre-treated with a mixture of K_2_S_2_O_8_ (0.5 g) and concentrated H_2_SO_4_ (5 mL, 98% w/w), at 90 °C, spiked with dyes, and using a two-stage dosage protocol (0.005 g each) to assess the material’s robustness in complex matrices^30^.

## Results and discussion

### Materials design

The AgFeO_2_/PPAC nanocomposite was designed as a multifunctional composite combining pomegranate-derived activated carbon(PPAC) with AgFeO_2_. The initial deep red colour of the raw pomegranate peel (Raw-PP) is shifted to black following the activation process (PPAC), eventually becoming brownish-black after the integration of AgFeO_2_. These shifts in colour serve as a preliminary indication of the structural and chemical modifications occurring at each stage of the synthesis.

The design of the nanocomposite aimed to anchor AgFeO_2_ nanoparticles onto the activated carbon framework, with glycerol acting as a structure-directing agent to promote phase purity. The resulting AgFeO_2_/PPAC nanocomposite maintains a clear magnetic response, which facilitates its separation from aqueous solutions using an external magnet. This visual evidence provides a consistent starting point for the detailed structural and morphological characterization that will be discussed in the following sections.

## Characterization

### FTIR spectra

#### FTIR spectral analysis of (PP), (PPAC), and AgFeO_2_/PPAC nanocomposite

**Fig. 3:**
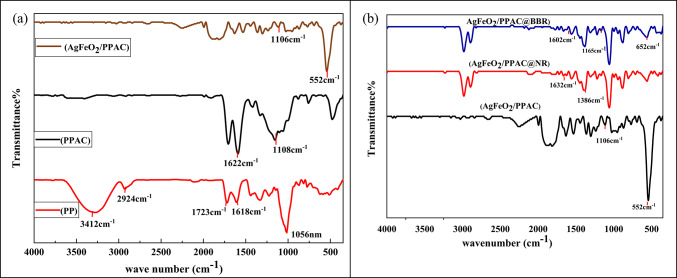
(**a**) FTIR spectra of PP, PPAC, and AgFeO_2_/PPAC nanocomposite, (**b**) FTIR spectra of AgFeO_2_/PPAC, AgFeO_2_/PPAC@NR and AgFeO_2_/PPAC@BBR -loaded dyes.

The FTIR spectra of raw pomegranate peel (PP), activated carbon (PPAC), and the AgFeO_2_/PPAC nanocomposite indicate changes in surface functional groups during the transformation from biomass to carbon-based composite in Fig. 3a. The PP spectrum shows characteristic bands at 3412 cm^−1^ (O–H stretching), 1723 cm^−1^ (C=O), at 1618 cm^−1^ (aromatic C=C), and 1056 cm^−1^ (C–O), confirming its lignocellulosic nature^48^.

After activation, the PPAC spectrum exhibits reduced intensity of O–H groups and enhanced C=C stretching around 1622 cm^−1^, indicating increased aromaticity and carbonization. The persistence of a C–O stretching band near 1108 cm^−1^ proves the phosphorylation process on the carbon surface^28^. For AgFeO_2_/PPAC, the O–H band exhibits a slight shift, suggesting new surface interactions. A notable increase in intensity and broadening in the 1602–1632 cm^−1^ regions indicates enhanced conjugation and surface restructuring. A band observed at ~ 552 cm^−1^ is attributed to metal–oxygen (Fe–O) vibrations, suggesting the presence of a metal oxide phase^48^. The low-frequency region (600–450 cm^−1^) further supports metal–oxygen bonding. These observations are consistent with the formation of AgFeO_2_ as proved by XRD analysis. This surface modification disturbs the electronic environment of the carbon, creating new active sites that are responsible for the enhanced dye adsorption capacity detected in the nanocomposite^49^.

#### FTIR of AgFeO_2_/PPAC nanocomposite—loaded dyes

The FTIR spectra provide information about the interaction between dye molecules and the AgFeO_2_/PPAC surface. In Fig. 3b For the cationic Neutral Red, new bands observed in the range of 1600–1630 cm^−1^ and 1380–1400 cm^−1^ are attributed to C=N and C–N vibrations of the dye structure, indicating its presence on the nanocomposite surface. A decrease in the intensity of the broad O–H band around 3400 cm^−1^ suggests involvement of surface hydroxyl groups in the interaction. These observations may suggest possible electrostatic attraction, hydrogen bonding, and π–π interactions between the dye molecules and the carbon matrix^50^.

The FTIR spectrum of the anionic Brilliant Blue R shows a new band in the 1120–1180 cm^−1^ region corresponds to S=O stretching of sulfonate groups, approving adsorption of the dye onto the surface. Another distinct band near 1600 cm^−1^ is associated with aromatic C=C structures. Furthermore, the attenuation of the nanocomposite own –OH and C=O bands indicates possible involvement of these groups in dye interaction. The appearance of bands between 620 and 680 cm^−1^, attributable to S–O bending and aromatic deformations, offers further proof of the dye’s presence on the surface^51^. Overall, the FTIR results indicate that both dyes are successfully adsorbed onto the nanocomposite surface through electrostatic attraction with the metal sites for the anionic BBR, and a combination of electrostatic and hydrogen bonding with surface oxygen groups for the cationic NR^52,53^.

#### SEM

**Fig. 4:**
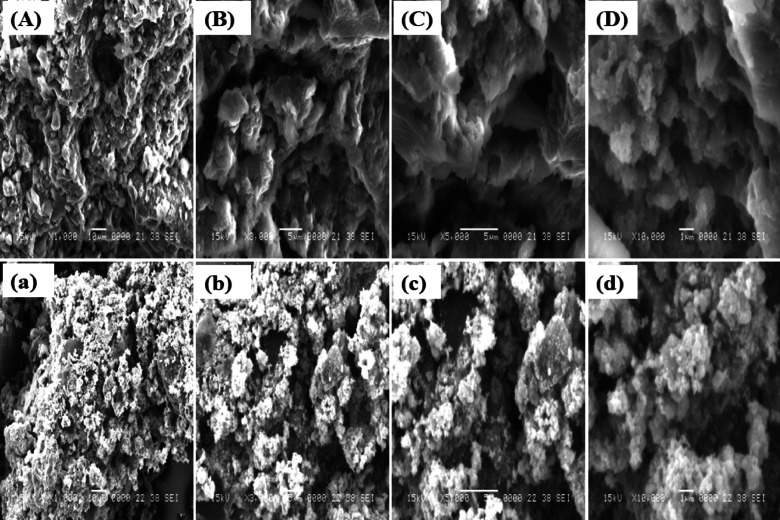
(**A**–**D**) SEM images of activated carbon (PPAC) and (**a**–**d**) SEM images of AgFeO_2_/PPAC nanocomposite at magnifications of 1000 × 3000 × , 5000 × and 10,000 × , respectively.

Scanning Electron Microscopy (SEM) analysis was carried out at different magnifications (1000×, 3000×, 5000×, and 10000×) to examine the surface morphology of the prepared materials, as seen in Fig. 4. The pristine activated carbon (PPAC) exhibits a rough and highly porous structure with well-developed channels and irregular pore walls, which are characteristic of biomass-derived activated carbon. After hydrothermal treatment, the morphology of the AgFeO_2_/PPAC nanocomposite shows noticeable changes. Bright nano-sized particles are observed on the carbon surface, indicating the presence of an inorganic phase. These particles are distributed over the porous structure, with a tendency to form localized aggregates, particularly along pore edges, leading to partial coverage of the carbon surface^54,55^.

This morphology is consistent with previously reported AgFeO_2_ materials, where particles typically appear as aggregated clusters rather than uniformly dispersed structures^56^. Despite this modification, the porous carbon framework remains visible, confirming that PPAC acts as a supporting matrix.

#### EDX

**Fig. 5:**
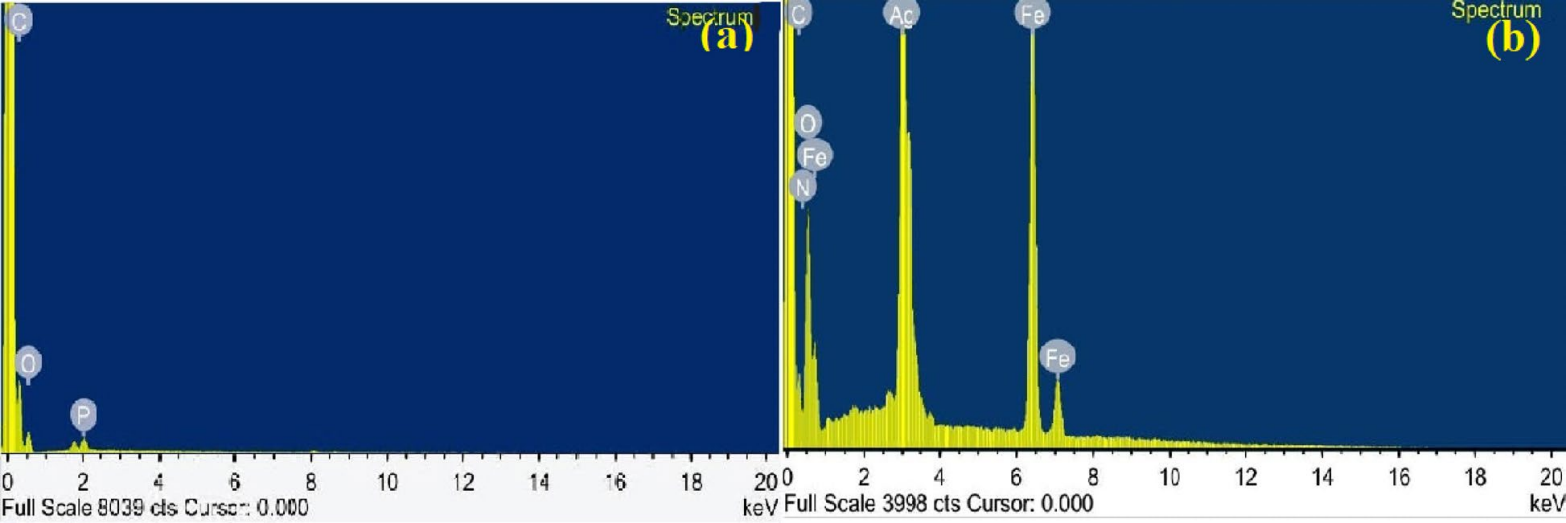
EDX spectrum of (**a**) activated carbon (PPAC) and (**b**) AgFeO_2_/PPAC nanocomposite.

This morphological observation is conclusively validated by Energy-Dispersive X-ray (EDX) spectroscopy, Fig. 5. The spectrum of the PPAC shows only dominant carbon and a minor oxygen signal. In contrast, the AgFeO_2_/PPAC nanocomposite exhibits prominent new peaks for silver (Ag) and iron (Fe), alongside a strengthened oxygen peak and a concurrent reduction in the carbon intensity. EDX elemental mapping confirms the homogeneous spatial distribution of Ag, Fe, O, and C, further supporting the successful formation of a delafossite AgFeO_2_ composite rather than a physically mixed or bimetallic system^57,58^.

The x-axis represents energy (keV) and the y-axis represents counts (intensity of detected X-rays).

#### X-ray diffraction (XRD) analysis

**Fig. 6:**
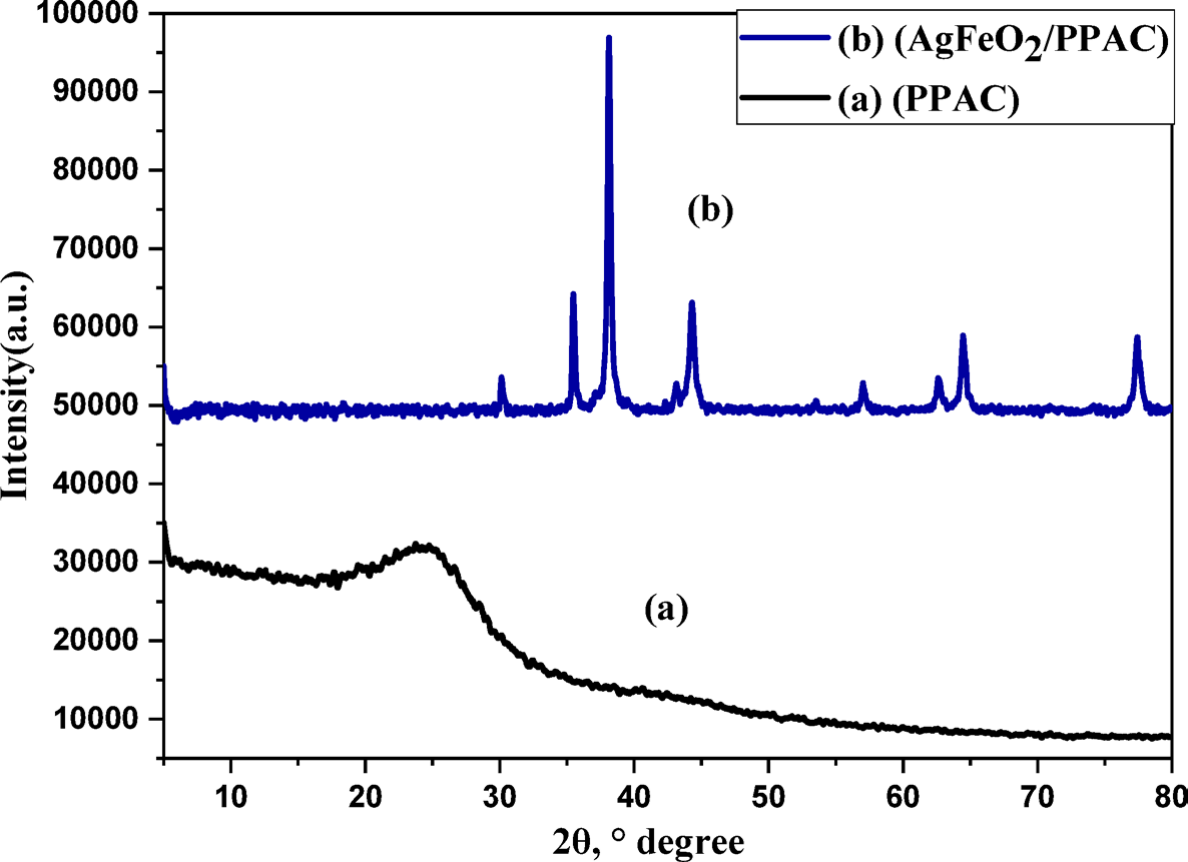
XRD patterns of (**a**) PPAC, and (**b**) AgFeO_2_/PPAC nanocomposite.

The X-ray diffraction (XRD) patterns provide further insight into the structural properties of the materials in Fig. 6. The XRD pattern of PPAC exhibits a broad diffraction peak centered at approximately 2θ ≈ 24°, which is characteristic of amorphous carbon and corresponds to the (002) plane of graphitic structures. The broad nature of this peak indicates a disordered arrangement of carbon layers, typical for activated carbon derived from biomass.

For the AgFeO_2_/PPAC nanocomposite, additional diffraction peaks are observed at approximately 2θ ≈ 32°, 38°, 46°, 55°, and 65°. These peaks are consistent with the reported diffraction pattern of rhombohedral delafossite AgFeO_2_. The presence of these reflections indicates the formation of a crystalline AgFeO_2_ phase within the composite^59^.

The characteristic carbon peak becomes less pronounced in the composite pattern. This can be attributed to the amorphous nature of PPAC and the overlap with the more intense diffraction peaks of the crystalline AgFeO_2_ phase. No distinct diffraction peaks corresponding to metallic silver were observed. Due to the one-step hydrothermal synthesis approach, AgFeO_2_ was formed in situ on the carbon surface rather than being prepared as a separate phase. However, the agreement between the observed diffraction peaks and previously reported data supports the successful formation of the AgFeO_2_ phase within the composite^56^.

#### BET analysis

The surface area of both PPAC and AgFeO_2_/PPAC nanocomposite was imposed on nitrogen adsorption–desorption isotherms. The BET analysis shows that exhibit linear behavior within the selected relative pressure range, with correlation coefficients close to unity (Fig. S1). As shown in Table 1, PPAC displays a higher pore volume (214.73 cm^3^ g^−1^) and a larger specific surface area (935 m^2^ g^−1^). After modification, the AgFeO_2_/PPAC nanocomposite shows reduced values, with a pore volume of 181.2 cm^3^ g^−1^ and a surface area of 789 m^2^ g^−1^. Both samples exhibit high BET constant (C) values (674 for PPAC and 660 for AgFeO_2_/PPAC), indicating strong interactions between the adsorbent surface and nitrogen molecules in the first adsorption layer^60^. The decrease in surface area after modification can be attributed to the partial coverage of PPAC pores by AgFeO_2_ nanoparticles, which limits the accessibility of nitrogen molecules during adsorption. Despite this reduction, the nanocomposite retains a high surface area, which remains suitable for adsorption applications^61^.

**Table 1:** The BET analysis of PPAC and AgFeO_2_/PPAC nanocomposite.

Parameters	PPAC	AgFeO_2_/PPAC
Pore volume v_m_	214.7 cm^3^(STP)·g^−1^	181.2 cm^3^(STP)·g^−1^
Surface area S_BET_	935 m^2^·g^−1^	789 m^2^·g^−1^
Constant C_BET_	674	660

#### TGA

**Fig. 7:**
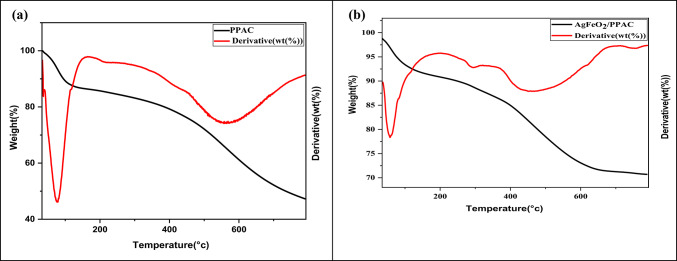
Thermal analysis of (**a**) PPAC and (**b**) AgFeO_2_/PPAC nanocomposite.

Thermogravimetric analysis (TGA) revealed distinct thermal degradation profiles for the PPAC and modified (AgFeO_2_/PPAC) materials, highlighting the effect of AgFeO_2_ incorporation, as shown in Fig. 7. The PPAC exhibited a three-stage decomposition behavior. An initial mass loss of approximately 13% below 120 °C is attributed to the removal of physically adsorbed moisture. This was followed by a second mass loss of about 8% in the intermediate temperature range, corresponding to the decomposition of labile oxygen-containing surface functional groups. A further mass loss of around 27% at higher temperatures reflects the progressive degradation of the carbon framework, resulting in a final residue consistent with the observed curve^62^. In contrast, the modified nanocomposite (AgFeO_2_/PPAC) displayed a more stable and simplified two-stage degradation pattern. A mass loss of approximately 9% at low temperature is associated with moisture removal. This is followed by a broader decomposition step with a total mass loss of about 20% over the higher temperature range, indicating improved structural stability and reduced thermal degradation. The higher remaining mass confirms the successful incorporation of the thermally stable AgFeO_2_ phase and demonstrates its role in enhancing the overall thermal stability of the carbon matrix^63^. This not only confirms the presence of the inorganic component but also suggests that the modification enhances the overall thermal stability of the carbon framework.

### Magnetic property and separation performance

**Fig. 8:**
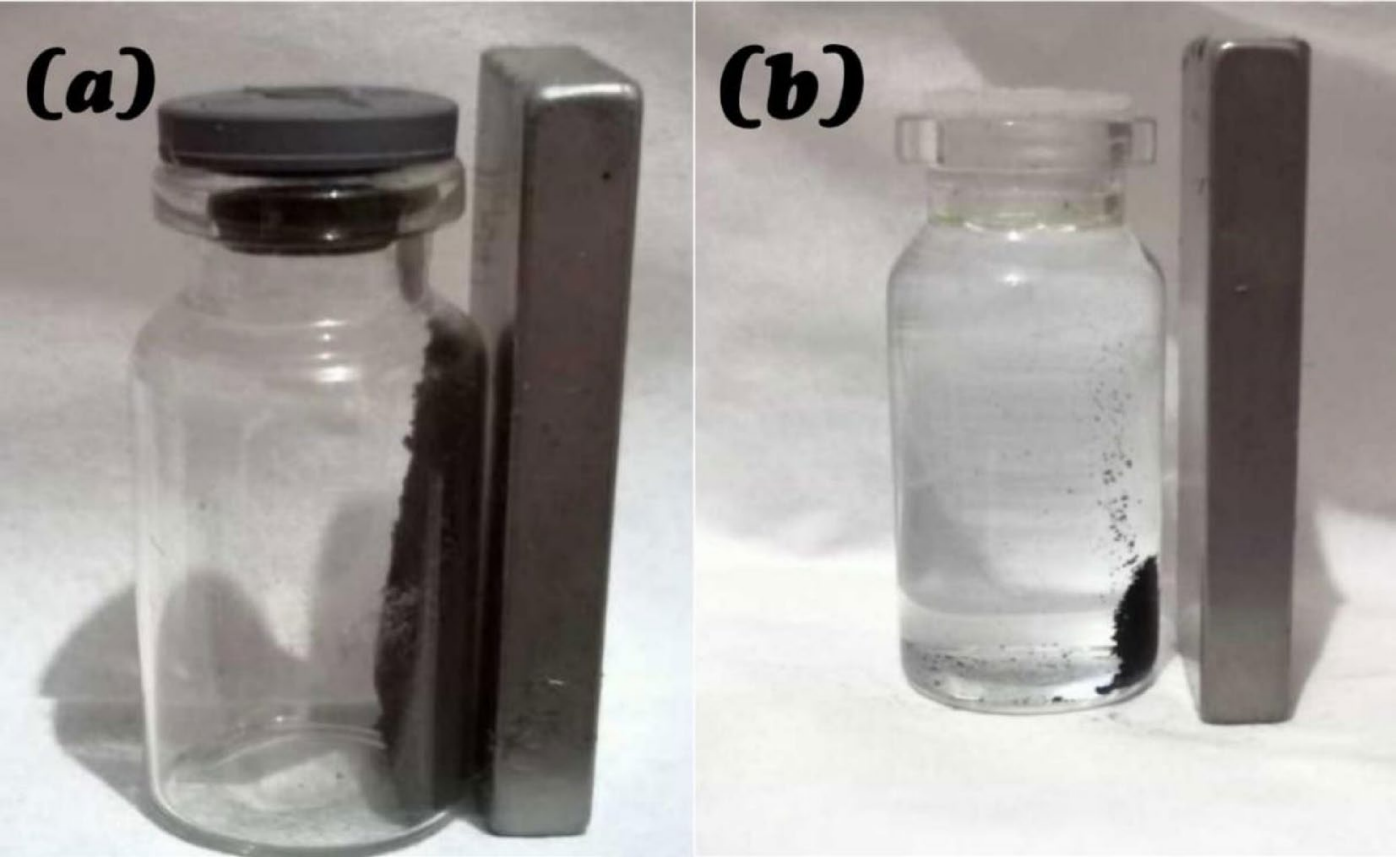
Separation of AgFeO_2_/PPAC nanocomposite in the dry form and in distilled water under an external magnetic field.

The AgFeO_2_/PPAC nanocomposite exhibits an effective magnetic response that facilitates its recovery after photocatalytic treatment. As demonstrated in Fig. 8, the photocatalyst can be readily separated both in the dry state and when dispersed in distilled water using an external magnet. This magnetic behaviour originates from the incorporation of Fe^3+^ ions within the delafossite AgFeO_2_ lattice rather than from magnetic iron oxide impurities. The magnetic-assisted separation allows the catalyst to be recovered, reused, and handled without filtration or centrifugation, enhancing its practical applicability for repeated wastewater treatment cycles. After removal of the external magnet, the separated AgFeO_2_ /PPAC particles lose magnetic alignment and spontaneously redisperse in the solution. This redispersion occurs due to Brownian motion and liquid convection, confirming a reversible and weak magnetic response^64^.

**Fig. 9:**
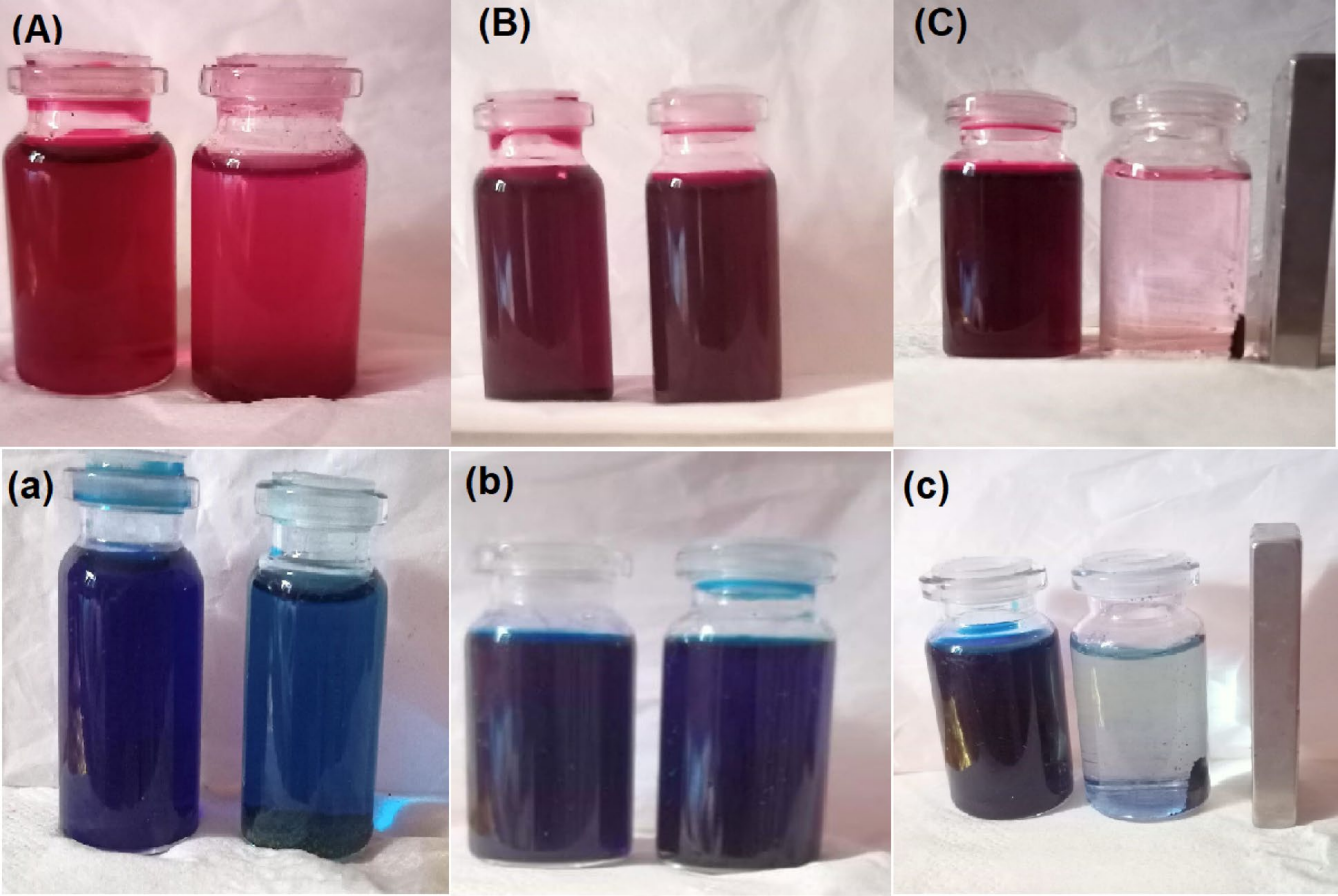
The digital photographs of (NR) and (BBR) using (**A**, **a**) pomegranate peel powder (pp), (**B**, **b**) Activated carbon (PPAC), and (**C**,** c**) AgFeO_2_/PPAC nanocomposite collected with external magnet.

Furthermore, the dye uptake experiments (Fig. 9A–C) provide a qualitative comparison of the materials. While Raw-PP and PPAC show limited adsorption (faint color changes), the AgFeO_2_/PPAC nanocomposite demonstrates efficient removal, resulting in a nearly colorless solution. The rapid separation using an external magnet confirms the composite’s magnetic properties and its potential for easy recovery in practical applications.

### Adsorptive photocatalytic studies

#### Point of zero charge (pH_PZC_)

The pH_PZC_ of the AgFeO_2_/PPAC nanocomposite was determined to be 6.21 (as shown in Fig. S2). At this value, the net surface charge of the nanocomposite is neutral. At pH > 6.21, the surface functional groups deprotonate, rendering the composite surface negatively charged, which is theoretically favorable for the adsorption of cationic species (NR). Conversely, at pH < 6.21, the surface becomes positively charged due to protonation, enhancing its affinity for anionic molecules (B BR) through electrostatic attraction.

#### Effect of pH

**Fig. 10:**
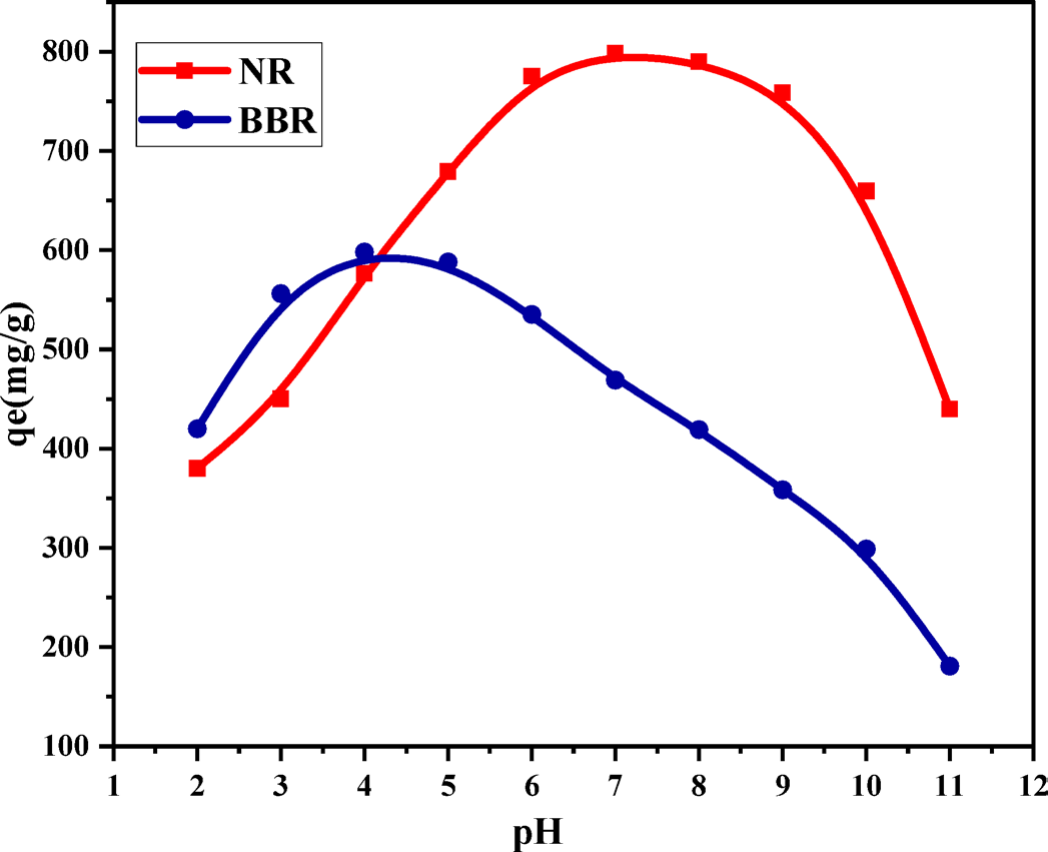
Effect of pH on the adsorptive photocatalytic degradation of (NR and BBR) dyes by AgFeO_2_/PPAC nanocomposite.

The influence of initial pH on the degradation of (NR, cationic) and (BBR, anionic) was evaluated through a range of 2–11 (Fig. 10).

For BBR (anionic Dye), the maximum removal efficiency was achieved at pH 4. This is consistent with the pH_PZC_ results, as the nanocomposite surface is positively charged at this acidic pH, leading to strong electrostatic attraction with the anionic BBR molecules. As the pH increased beyond 6.21, the removal efficiency declined significantly due to the electrostatic repulsion between the now negatively charged surface and the anionic dye^65^.

For NR (cationic Dye), the optimal removal was observed at pH 7. Although the surface becomes more negatively charged at higher pH values (favoring cationic NR), the observed decrease in efficiency at highly alkaline pH (above pH 9) can be attributed to the potential instability of the dye molecules or changes in the surface speciation of the photocatalyst^66,67^. The decrease at acidic pH (below pH 6) is due to the electrostatic repulsion between the positively charged surface and the cationic NR, alongside competition with H^+^ ions for active sites^50^.

#### Effect of AgFeO_2_/PPAC nanocomposite dose

The influence of AgFeO_2_/PPAC dose on dye degradation was studied from 0.0025 to 0.01 g. Both NR and BBR reached maximum removal at 0.005 g. At lower doses, limited active sites reduced adsorption efficiency. Increasing the dose beyond 0.005 g showed minimal improvement, indicating that surface sites became saturated and additional material did not significantly enhance removal as illustrated in Fig. S3^35^.

#### Effect of initial dye concentration and adsorption-degradation isotherms

**Fig. 11:**
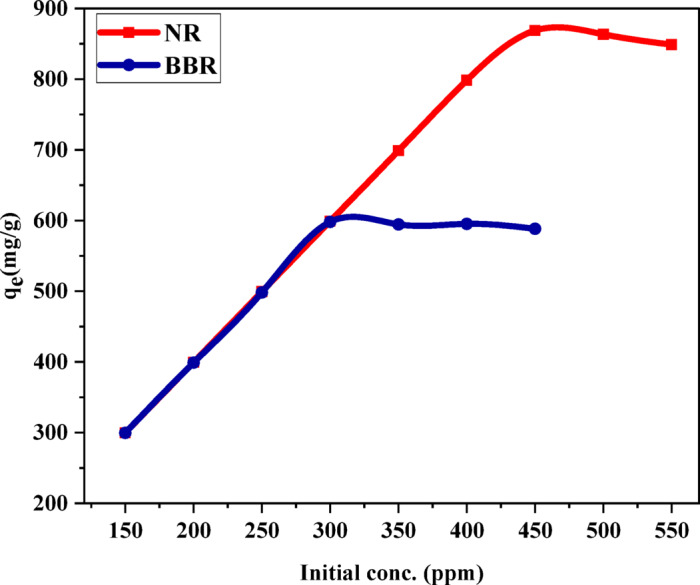
The effect of initial dye concentration on the degradation of (**a**) NR, (**b**) BBR by AgFeO_2_/PPAC nanocomposite.

The influence of initial dye concentration on the adsorption-photodegradation of NR and BBR was investigated using the AgFeO_2_/PPAC nanocomposite (Fig. 11). The study exposed concentrations ranging from 150 to 550 μg/mL for NR and 150 to 450 μg/mL for BBR. For NR, removal capacity increased gradually up to 400 μg/mL, followed by a marginal increase until a steady state was achieved. This trend reflects the progressive occupation of active sites, eventually leading to surface saturation. Accordingly, 400 μg/mL was identified as the optimal concentration for NR, achieving an uptake (q_e_) of 798.5 mgg^−1^ and a removal efficiency (R%) of 99.8%. BBR reached its peak capacity at 300 μg/mL (q_e_ = 598 mg.g^−1^, R% = 99.67%), showing earlier saturation and more pronounced diffusion limitations than NR^68,69^.

**Fig. 12:**
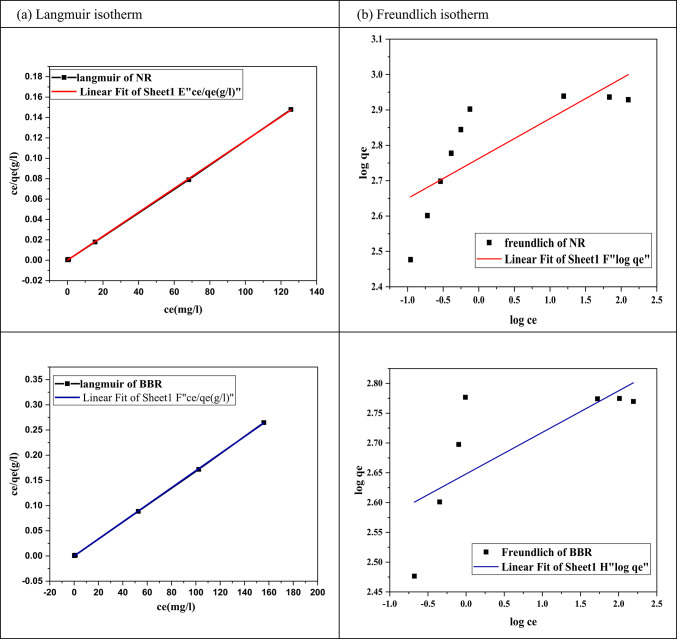
Adsorption**-**photodegradation isotherms for NR and BBR by AgFeO_2_/PPAC nanocomposite: (**a**) Langmuir isotherm model, (**b**) Freundlich isotherm model.

To further interpret these interactions, the data were fitted to Langmuir, Freundlich, Temkin, and D–R isotherm models (Table 2; Fig. 12). The Langmuir model provided the best fit, yielding exceptionally high correlation coefficients (R^2^ = 0.9999) for both dyes. This strong alignment suggests that the remediation process primarily follows a monolayer pattern on relatively uniform active sites. The calculated maximum monolayer capacities (q_max_) were 854.7 mg.g^−1^ for NR and 591.72 mg.g^−1^ for BBR, while the low separation factors (R_L_) confirmed that the process is highly favorable. Conversely, the Freundlich and Temkin models showed poor correlation (R^2^ < 0.70), indicating that multilayer interaction is not the dominant mechanism. Additionally, the D–R model yielded mean free energy values of 22.8 kJ/mol for NR and 32.1 kJ/mol for BBR, which are consistent with a chemisorption-driven pathway.

**Table 2:** Parameters of Langmuir, Freundlich, Temkin, and D–R isotherm models for adsorption-catalytic degradation of NR and BBR dyes onto AgFeO_2_/PPAC nanocomposite.

Langmuir isotherm								
System	q_max_ (mg/g)	K_L_	R_L_	R^2^	${\mathrm{x}}^{{2}}$	SSE	MSE	Hybrid
AgFeO_2_/PPAC @NR	854.7	22.3	1.12 × 10^–4^	0.99991	859.3	734.6	816.3	278.2
AgFeO_2_/PPAC @BBR	591.7	68.2	4.9 × 10^–5^	0.99995	221.8	131.9	187.98	79.9

Freundlich isotherm							
System	N	K_F_	R^2^	${\mathrm{x}}^{{2}}$	SSE	MSE	Hybrid
AgFeO_2_/PPAC @NR	8.9	578.8	0.62	1425.4	1417.4	1574.1	497.3
AgFeO_2_/PPAC @BBR	14.3	444.6	0.55	293.5	184.8	264.3	108.7

System	Temkin isotherm constants		
	B (j/mol)	K_T_ (L/g)	R^2^
AgFeO_2_/PPAC @NR	37.5	1.7 × 10^4^	0.7
AgFeO_2_/PPAC @BBR	79.2	1.9 × 10^6^	0.6

System	D-R isotherm constants		
	K_D-R_	E (kJ/mol)	R^2^
AgFeO_2_/PPAC @NR	9.7 × 10^–10^	22.8	0.7
AgFeO_2_/PPAC @BBR	4.9 × 10^–10^	32.1	0.6

While biomass-derived carbons like PPAC are inherently heterogeneous, the high degree of linearity observed suggests that the AgFeO_2_ active sites are well-distributed across the framework. This distribution creates an effectively homogeneous environment for the degradation-assisted recovery of surface sites. These results indicate that while higher concentrations increase the mass transfer driving force, the overall efficiency is ultimately governed by the availability of photo-active centers and the balance between surface adsorption and light-induced mineralization^50,70^.

#### Effect of shaking time and photocatalytic kinetic studies

**Fig. 13:**
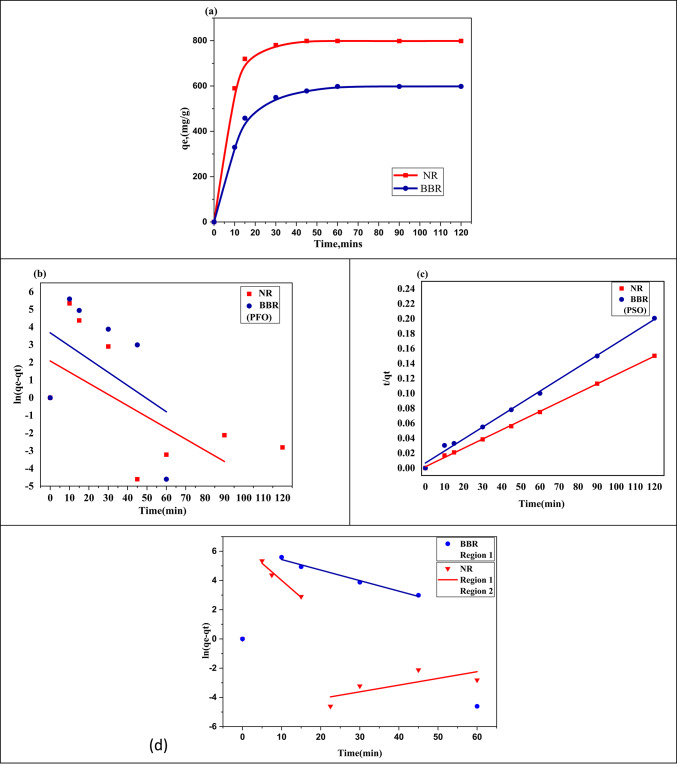
(**a**) Effect of shaking time on adsorption-photodegradation of NR and BBR by AgFeO_2_/PPAC, (**b**) Pseudo-1st-order model (**c**) Pseudo-2nd-order model and (**d**) Segmented PFO for the dynamic removal of NR and BBR dyes over the AgFeO_2_/PPAC nanocomposite surface.

The effect of contact time on the removal of NR and BBR was investigated over 10–120 min. The maximum degradation was achieved at 45 min for NR and 60 min for BBR in Fig. 13a, indicating rapid interaction between the dye molecules and the active sites of the AgFeO_2_/PPAC nanocomposite. The variation in contact time reflects the gradual approach toward equilibrium and provides insight into the kinetic behaviour governing the removal process^71,72^. To further evaluate the degradation mechanism, the experimental data were analyzed using pseudo-first-order (PFO), pseudo-second-order (PSO), and Weber–Morris intra-particle diffusion models, as presented in Fig. 13b–d and Table 3.

**Table 3:** Kinetic parameters for NR and BBR dyes degradation using AgFeO_2_/PPAC nanocomposite.

Experimental data of Pseudo first order kinetic model (PFO)							
System	q_e_(mg/g)	K_1_(min^−1^)	R^2^	${\mathrm{x}}^{{2}}$	SSE	MSE	Hybrid
AgFeO_2_/PPAC @NR	14.7	0.06	0.4	263,754.8	387.6	553.5	101.8
AgFeO_2_/PPAC @BBR	97	0.01	0.3	14,158.8	137.5	196.3	496.1

Experimental data of Segmented Pseudo first order kinetic model (PFO)				
	Region	Time Range (min)	K_1_(min^−1^)	R^2^
AgFeO_2_/PPAC @NR	Initial	10–30	0.12	0.97
	Final	45–120	0.02	0.53
AgFeO_2_/PPAC @BBR	Initial	10–45	0.07	0.98

Experimental data of Pseudo second order kinetic model (PSO)							
System	q_e_(mg/g)	K_2(min_^−1^_)_	R^2^	${\mathrm{x}}^{{2}}$	SSE	MSE	Hybrid
AgFeO_2_/PPAC @NR	806.5	0.0009	0.9992	68.7	55.3	79.8	18.9
AgFeO_2_/P PAC @BBR	617.3	0.0004	0.9962	186.8	115.98	16.6	63.9

System	Experimental data of Linear IPD	
	k_diff_ (mg.g^−1^.min^−1^)	R^2^
AgFeO_2_/PPAC @NR	188.1	0.999
AgFeO_2_/PPAC @BBR	114.02	0.994

System	Experimental data of Linear Elovich		
	${\upalpha }$_e_ (mg/g.min)	q_e_(mg/g)	R^2^
AgFeO_2_/PPAC @NR	6.8 × 10^4^	765.8	0.7
AgFeO_2_/PPAC @BBR	492.6	574.5	0.8

The kinetic analysis revealed that the PSO model provided the best fit for both dyes, with high correlation coefficients of R^2^ = 0.9992 for NR and 0.9962 for BBR. The calculated equilibrium capacities reached 806.5 mg/g for NR and 617.3 mg/g for BBR, showing close agreement with the experimental values and indicating that the overall process is predominantly governed by chemisorption through active-site interactions. In contrast, the conventional PFO model showed poor agreement, with low R^2^ values of 0.4 for NR and 0.3 for BBR, indicating that simple physical adsorption alone could not adequately describe the degradation pathway^50,73^.

To gain deeper insight into the kinetic stages, a segmented two-region linear regression was applied to the PFO model (Fig. 13d). The analysis demonstrated two distinct regions for NR. The first stage exhibited a rapid adsorption rate (k_1,1_ = 0.12 min^−1^, R^2^ = 0.97), corresponding to the immediate migration of dye molecules toward the highly accessible surface sites, followed by a slower second stage (k_1,2_ = 0.02 min^−1^) attributed to gradual pore diffusion and surface saturation. For BBR, the first stage also showed fast kinetics (k_1,1_ = 0.07 min^−1^, R^2^ = 0.98), while the second stage became negligible since near-complete removal was achieved within the initial period. These findings indicate that the removal process proceeds through multiple sequential steps involving rapid surface adsorption followed by slower diffusion within the porous structure.

The Weber–Morris intra-particle diffusion model further confirmed the multi-step degradation mechanism. For NR, the plot exhibited excellent linearity (R^2^ = 0.99) with two distinct diffusion regions. The initial stage showed a very high diffusion constant (k_id,1_ = 188.1 mg g^−1^ min^−0.5^), reflecting rapid film diffusion and strong surface affinity, whereas the second stage displayed a much lower value (k_id,2_ = 2.5 mg g^−1^ min^−0.5^) as equilibrium was approached. Similarly, BBR exhibited a multi-linear profile (R^2^ = 0.99), with k_id,1_ = 114 mg g^−1^ min^−0.5^ during the fast initial migration stage and k_id,2_ = 8.1 mg g^−1^ min^−0.5^ during the slower intra-particle diffusion stage. The near-zero intercept values for both dyes suggest minimal boundary layer resistance and highlight the synergistic contribution of rapid chemisorption and pore diffusion during the removal process.

The Elovich kinetic model was also applied to evaluate the heterogeneous chemisorption behaviour of the AgFeO_2_/PPAC surface as obtained in Table 3. The calculated removal capacities reached 765.8 mg/g for NR and 574.5 mg/g for BBR, with initial adsorption rates (α_e_) of 6.8 × 10^4^ and 492.6 mg g^−1^ min^−1^, respectively. The moderate correlation coefficients (R^2^ = 0.7 for NR and 0.8 for BBR) indicate that the Elovich model partially describes the removal mechanism through surface heterogeneity and chemisorption behaviour. Despite the additional insight obtained from segmented PFO fitting and diffusion analysis, the PSO model remained the most reliable descriptor of the overall adsorption–photodegradation mechanism, confirming that the rate-limiting step is primarily controlled by chemical interactions involving electron sharing or exchange between the dye molecules and the AgFeO_2_/PPAC surface.

#### Effect of temperature and thermodynamic studies

The influence of temperature on the remediation of NR and BBR was evaluated between 30 and 65 °C (Fig. S4). For both dyes, an increase in temperature led to enhanced uptake, suggesting an endothermic nature for the process. This improvement at higher temperatures can be attributed to increased molecular mobility and the facilitation of stronger interactions between the dye molecules and the AgFeO_2_/PPAC surface.

The thermodynamic parameters, summarized in Table 4, further characterize the energetic nature of the system. The negative values of Gibbs free energy (ΔG°) across all tested temperatures indicate the spontaneous nature of the process. For NR, ΔG° values ranged from − 17.7 kJ/mol at 303 K to − 19.1 kJ/mol at 338 K, while for BBR, they ranged from − 16.6 kJ/mol to − 18.8 kJ/mol. The positive enthalpy changes (ΔH°) = 11.1 kJ/mol for NR and 17.9 kJ/mol for BBR) confirm the endothermic pathway. Additionally, the positive entropy changes (ΔS°) = 95.3 J/mol·K for NR and 113.7 J/mol·K for BBR) reflect an increase in randomness at the solid–liquid interface during the interaction. Collectively, these results suggest that the removal process is administrated by strong surface interactions, consistent with a chemisorption-driven mechanism where the rate is influenced by surface reaction kinetics^50,70^.

**Table 4:** Thermodynamic parameters for the adsorption of NR and BBR onto AgFeO_2_/PPAC nanocomposite.

System	T(^o^K)	KC	ΔG^o^ (kJ/mol)	ΔH°_ads_ (kJ/mol)	ΔS° (J/mol.K)
AgFeO_2_/PPAC @NR	303	1124.8	− 17.7	11.1	95.3
	318	1228.8	− 17.9		
	338	1950.8	− 19.1		
AgFeO_2_/PPAC @BBR	303	712.3	− 16.6	17.9	113.7
	318	757.5	− 16.7		
	338	1712.3	− 18.8		

### UV–Vis absorption spectra of single and binary systems

**Fig. 14:**
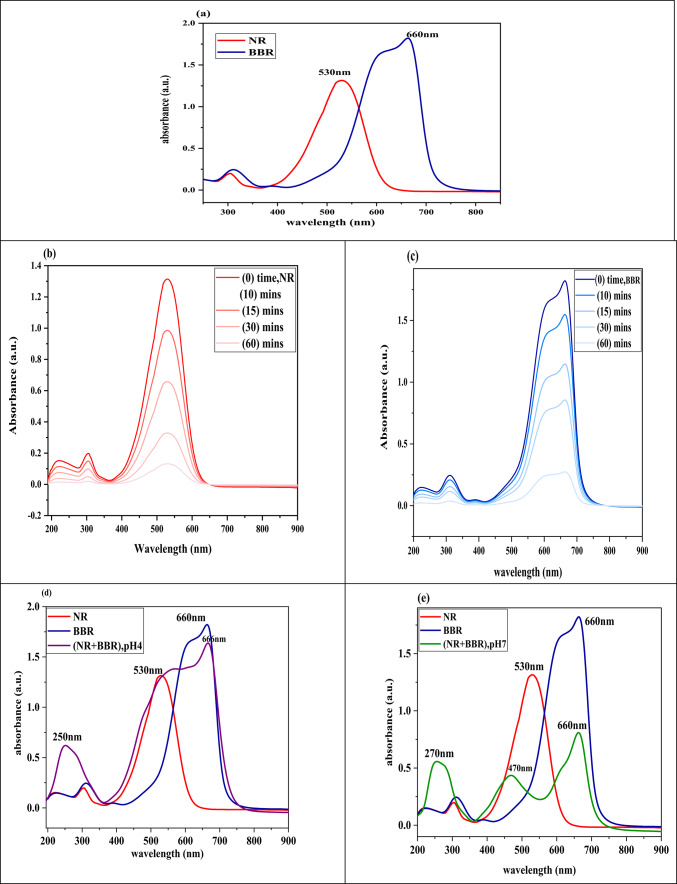

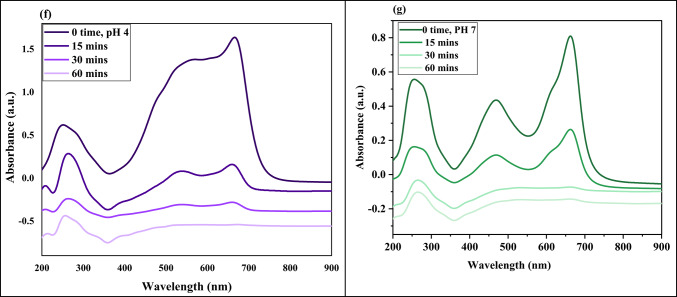
UV–Vis absorption spectra of (**a**) NR and BBR (**b**) degradation of NR at different time intervals, (**c**) degradation of BBR at different time intervals, (**d**) degradation of NR + BBR at pH4, (**e**) (NR + BBR at pH7) and (**f**, **g**) (NR + BBR at pH4), (NR + BBR at pH7) at different time intervals, respectively.

The UV–Vis absorption spectra for the individual dyes revealed characteristic λ_max_ values at 590 nm for (NR) and 660 nm for (BBR), as illustrated in Fig. 14a. Monitoring the absorbance over time showed a consistent decrease in peak intensity for both dyes, indicating the effective photocatalytic performance of the AgFeO_2_/PPAC nanocomposite. Particularly, the time-dependent measurements indicated that NR underwent faster degradation compared to BBR, as shown in Fig. 14b and c. In the binary system (Fig. 14d–g), the resulting spectra were not merely a linear outline of the individual components; instead, distinct changes in band shape and intensity were observed, pointing to competitive absorption and spectral overlap between the two chromophores.

The solution’s pH played a critical role in the optical behavior of the mixture, even before the addition of the photocatalyst. At pH 7, the solution appeared green, while shifting to pH 4 turned it purple. This transition is attributed to the pH-dependent chemical states of NR and BBR and their specific light absorption near 530 nm and 660 nm. Furthermore, the surface charge of the AgFeO_2_/PPAC nanocomposite characterized by a pH_pzc_ of approximately 6.2 significantly influenced the process. At pH 4, the positively charged surface favored the attraction of the anionic BBR molecules. Conversely, at pH 7, the surface became negatively charged, promoting the uptake of the cationic NR^79,80^.

Under visible light irradiation, the primary absorption peaks for both dyes decreased steadily. Significant remediation was observed within the first 15 min, with nearly complete decolorization by 60 min. This visual fading overlapped with the emergence of new, smaller peaks at lower wavelengths (near 250 nm at pH 4, and around 270 nm and 470 nm at pH 7). These new signals, along with the progressive loss of the main absorbance bands, suggest the breakdown of the dyes’ molecular structures into intermediate fragments^74,75^. The observed peak shifts and mutual suppression in the binary system further prove the competitive behavior of NR and BBR on the catalyst’s active sites during the simultaneous adsorption and photocatalytic degradation process.

#### Desorption and reusability studies

**Fig. 15:**
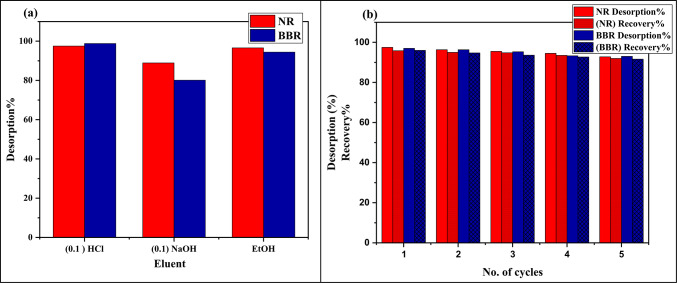
(**a**) Desorption of NR and BBR from AgFeO_2_/PPAC nanocomposite using different eluents, (**b**) Repeated five cycles of NR and BBR adsorption–desorption using HCl for both dyes as eluent, (*n* = 5).

The stability and reusability of the AgFeO_2_/PPAC nanocomposite were investigated through sequential desorption cycles. To evaluate the desorption capacity, 0.1 M HCl, ethanol, and 0.1 M NaOH were compared (Fig. 15a). Results showed that 0.1 M HCl yielded the highest desorption efficiency for both NR and BBR. This performance is likely associated with the protonation of surface functional groups in acidic media, which reduces the affinity between the dyes and the nanocomposite surface.

The reusability of the AgFeO_2_/PPAC nanocomposite was subsequently conducted over five cycles using 0.1 M HCl as the eluent (Fig. 15b). The summarized results in Table 5 indicate that the recovery percentages for NR decreased from 95.8 to 92.0% between the first and fifth cycles. Similarly, BBR recovery changed from 96.0 to 91.6% over the same period^40^. The obtained data indicate that the AgFeO_2_/PPAC nanocomposite maintains its operational stability over multiple cycles, which is attributed to the structural consistency of the carbon framework and the immobilized AgFeO_2_ phase under the investigated regeneration conditions.

**Table 5:** Repeated degradation of NR and BBR dyes using AgFeO_2_/PPAC nanocomposite.

Dye	Cycle	Desorption (%)	Recovery (%)
NR	1	97.5	95.8
	2	96.3	95.0
	3	95.5	94.8
	4	94.5	93.5
	5	92.8	92.0
BBr	1	97.0	96.0
	2	96.3	94.7
	3	95.3	93.6
	4	93.3	92.7
	5	93.0	91.6

#### The effect of ionic strength

**Fig. 16:**
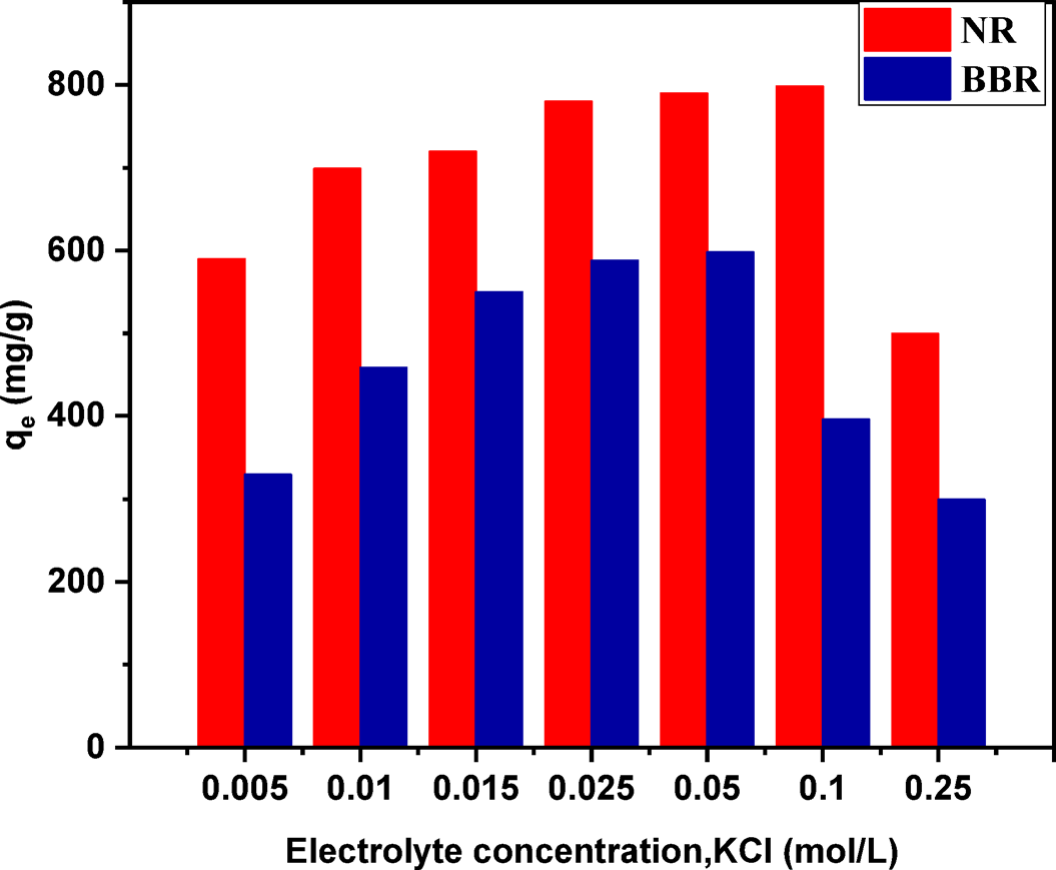
The effect of ionic strength (KCl with different conc.) on the degradation performance of AgFeO_2_/PPAC nanocomposite.

The influence of ionic strength on the performance of the AgFeO_2_/PPAC nanocomposite was evaluated using KCl concentrations ranging from 0.005 to 0.25 M (Fig. 16). The removal capacities were observed at 0.05 M KCl for BBR (q_e_ = 598 mg/g) and 0.10 M KCl for NR (q_e_ = 798.6 mg/g). Beyond these concentrations, a gradual decline in both q_e_ and removal efficiency was noted for both dyes. These observations suggest that moderate ionic strength may slightly enhance uptake by compressing the electrical double layer and reducing electrostatic repulsion, which facilitates the approach of dye molecules to the active sites.

However, at higher KCl concentrations, the reduction in capacity can be attributed to several factors. For the cationic NR, K^+^ ions compete for the negatively charged surface sites, while for the anionic BBR; Cl^−^ ions may apply a shielding effect on the positive centers of the nanocomposite. Furthermore, the high concentration of ions increases the screening of electrostatic attractions, which are essential for effective surface interaction^18^. This dual effect of competition and charge screening aligns with literature reports, confirming that while salt ions can initially assist in double-layer compression, excessive ionic strength ultimately interferes with the dye-adsorbent interactions by occupying or masking the available active sites.

### Applications

The feasibility of AgFeO_2_/PPAC nanocomposite for practical applications was examined by testing its performance across various aqueous matrices, including tap water, industrial wastewater (from Mahalla al-Kubra), and seawater (from Marsa Matrouh). The summarized results in Table 6 show high recovery rates ranging from 97.1 to 99.0% for both NR and BBR at different spiked levels (100 and 250 μg/mL). These findings indicate that the nanocomposite maintains its functional effectiveness despite the presence of potential interferences, such as high salinity or dissolved organic matter. While these spiked-matrix studies confirm the robustness and analytical reliability of the process, they provide a fundamental basis for future investigations into non-spiked, large-scale industrial systems. Ultimately, the consistent efficiency observed across these diverse water types supports the potential of AgFeO_2_/PPAC as a stable photocatalyst for remediation in varied environmental sites.

**Table 6:** Analytical results of photocatalytic degradation (μg/mL) of NR and BBR dyes in real water samples using AgFeO_2_/PPAC nanocomposite.

Type of dye	Wastewater samples type & location	Added (µg/ mL)	Found (µg/ mL)	Recovered (µg/ mL)	Recovery (%)
NR	Tap water (Mansoura university, Mansoura, Egypt)	0.00	0.00	0.00	0.00
		100	1.6	98.4	98.4
		250	3.1	246.9	98.8
	Wastewater (Industrial drain from the Textile Factory in Mahalla al-Kubra,	0.00	0.00	0.00	0.00
		100	2.1	97.9	97.9
		250	3.6	246.4	98.6
	Sea water (Marsa Matrouh, Egypt)	0.00	0.00	0.00	0.00
		100	1.4	98.6	98.6
		250	2.5	247.5	99.0
BBR	Tap water (Mansoura university, Mansoura, Egypt)	0.00	0.00	0.00	0.00
		100	1.9	98.1	98.1
		250	3.3	246.7	98.7
	Wastewater (Industrial drain from the Textile Factory in Mahalla al-Kubra	0.00	0.00	0.00	0.00
		100	2.9	97.1	97.1
		250	5.4	244.6	97.8
	Sea water (Marsa Matrouh, Egypt)	0.00	0.00	0.00	0.00
		100	1.6	98.4	98.4
		250	2.7	247.3	98.9

### Influence of visible light on NR and BBR photocatalytic degradation

**Fig. 17:**
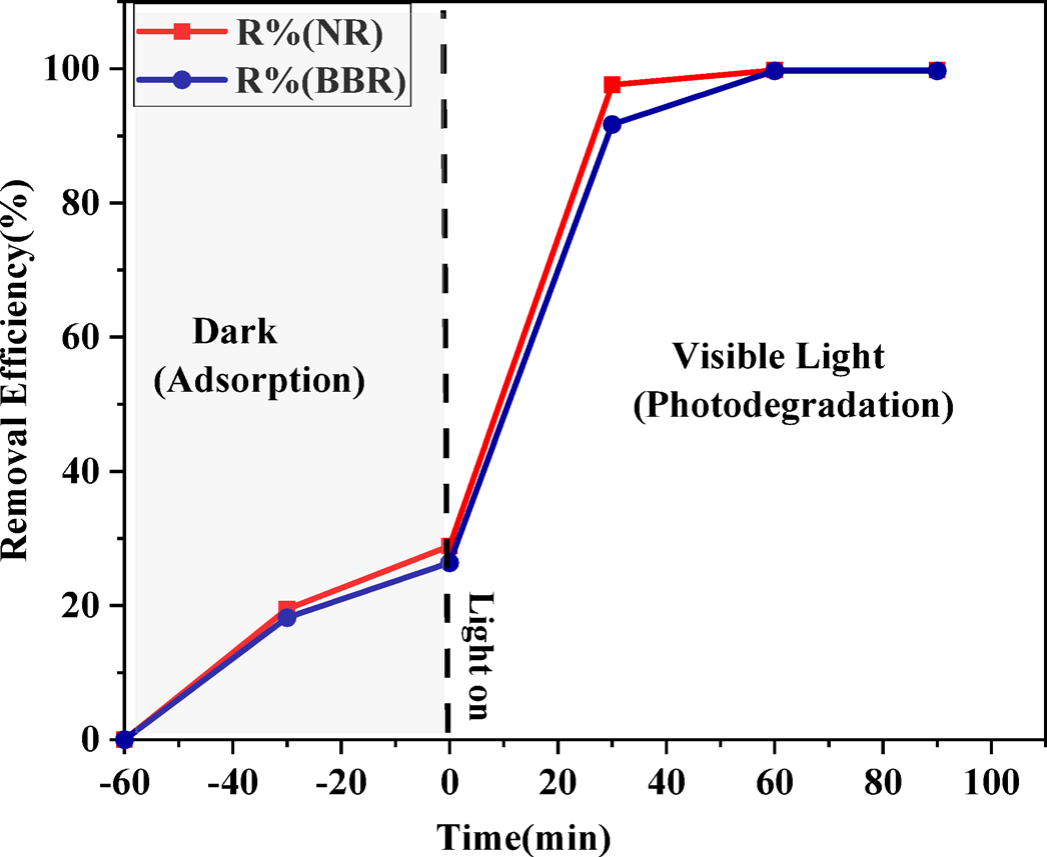
The effect of visible light with time on adsorptive-degradation of NR and BBR dyes using AgFeo_2_/PPAC nanocomposite.

The influence of visible light on the photocatalytic degradation of NR and BBR was evaluated through controlled experiments under dark and irradiated conditions (Fig. 17). During the initial 30-min dark phase, the removal efficiencies reached 28.9% for NR and 26.4% for BBR, representing the adsorption equilibrium established on the AgFeO_2_/PPAC surface. Upon exposure to visible light, the removal percentages increased to 99.1% and 89.9%, respectively.

The progressive decolorization of the solutions, confirmed by the disappearance of characteristic absorbance peaks in the UV–Vis spectra and the visual transparency shown in Fig. 9c, suggests the chemical breakdown of the dye’s chromophoric structures. Furthermore, the high recovery percentages (> 91%) maintained over five consecutive cycles indicate that the active sites are effectively regenerated through the degradation of organic molecules rather than a cumulative physical sequestration. These results support a synergistic mechanism where the PPAC framework facilitates initial surface proximity, while the AgFeO_2_ phase drives the light-induced degradation process.

### Proposed interaction between dye molecules (NR&BBR) and photocatalyst (AgFeO_2_/PPAC)

A schematic illustration of the proposed adsorption–photocatalytic pathway over AgFeO_2_/PPAC is presented in Fig. S5. The removal process is assumed to proceed through two coupled stages, involving initial adsorption of dye molecules onto the nanocomposite surface followed by photocatalytic degradation under visible light irradiation. Similar adsorption-assisted photocatalytic behavior has been reported for heterostructured photocatalysts, where surface interactions play a key role in promoting dye degradation.

**Fig. 18:**
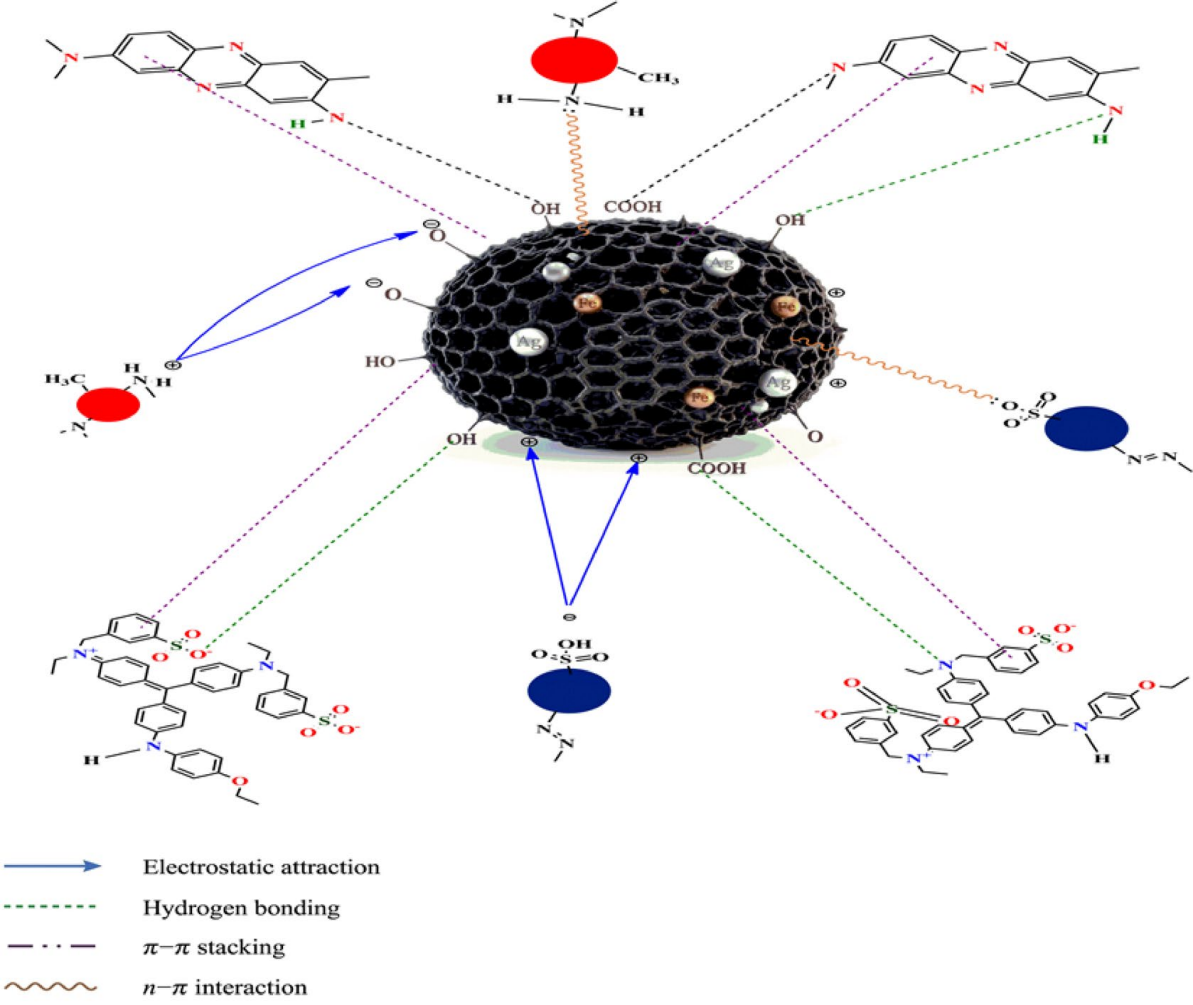
Proposed molecular-level interactions governing adsorption of NR and BBR dyes on AgFeO_2_/PPAC surface.

The adsorption behavior of NR and BBR could be associated with the porous carbon framework of PPAC together with the surface functional groups of AgFeO_2_/PPAC. Based on the pH_PZC_ results, FTIR spectra of AgFeO_2_/PPAC before and after adsorption, and the molecular structures of the dyes, several interactions may contribute to dye uptake. These interactions are proposed as possible adsorption pathways rather than definitive mechanisms. Electrostatic attraction may occur between the charged dye molecules and oppositely charged active sites on the nanocomposite surface depending on solution pH. Hydrogen bonding may also take place between surface hydroxyl groups and heteroatoms (N,O) present in NR and BBR molecules. In addition, the aromatic domains of PPAC can promote π–π stacking interactions with the aromatic rings of the dye structures, enhancing adsorption affinity. Furthermore, lone pair electrons associated with nitrogen- or oxygen-containing groups may participate in n–π interactions with the conjugated surface of the nanocomposite^76^. Figure 18 summarizes the possible molecular-level interactions governing dye adsorption on AgFeO_2_/PPAC.

Under visible light irradiation, AgFeO_2_ generates electron–hole pairs that participate in the formation of reactive oxygen species, including ·OH and ·O_2_^−^ radicals, which subsequently attack the adsorbed dye molecules and promote their degradation. The pre-adsorption of NR and BBR near the active photocatalytic sites is expected to facilitate interfacial charge transfer and improve degradation efficiency. Similar synergistic effects between adsorption and photocatalysis have been described for organic dye degradation over semiconductor heterostructured^61,77^.

### Proposed adsorption–photocatalytic mechanism of NR and BBR dyes using AgFeO_2_/PPAC nanocomposite

The removal of NR and BBR using the AgFeO_2_/PPAC nanocomposite is proposed to proceed through a synergistic adsorption–photocatalytic process. At the initial stage, dye molecules are adsorbed onto the porous carbon surface, which offers a high surface area and facilitates the accumulation of pollutants near the active sites. Upon visible light irradiation, AgFeO_2_ is assumed to generate electron–hole pairs (e^−^/h^+^), while the carbon matrix may contribute to improved charge separation and electron transfer, thereby limiting charge recombination.

The generated charge carriers are expected to react with dissolved oxygen and water molecules, leading to the possible formation of reactive oxygen species (ROS), mainly hydroxyl radicals (·OH) and superoxide radicals (·O_2_^−^). These reactive species are presumed to attack the chromophoric structures of the dyes, particularly the phenazine ring of NR and the triarylmethane structure of BBR, resulting in gradual oxidative fragmentation into smaller intermediates that can finally mineralize into CO_2_ and H_2_O.

FTIR observations provide supportive evidence for dye interaction and possible structural transformation during the degradation process. The appearance of a band near 1682 cm^−1^ may be attributed to C=O stretching vibrations associated with oxidized intermediate species. Meanwhile, the reduced intensity around 1165 cm^−1^ may indicate alterations in sulfonate (–SO_3_^−^) groups of BBR and C–N bonds of NR. Changes detected in the fingerprint region near 652 cm^−1^ also suggest modification of aromatic structures during treatment.

**Fig. 19:**
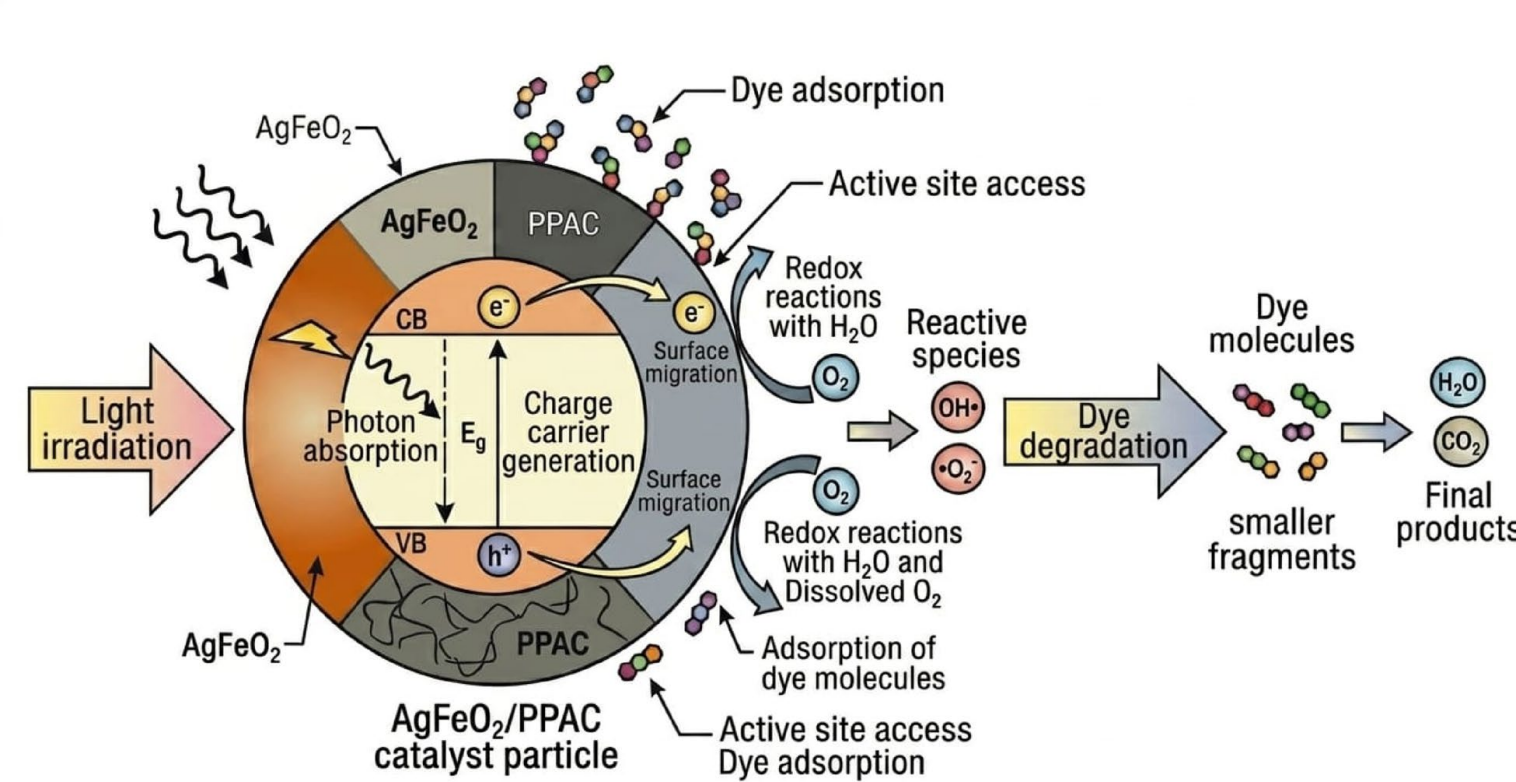
The proposed mechanism of photocatalytic degradation of NR and BBR using AgFeO_2_/PPAC nanocomposite.

Based on these findings, the overall mechanism is proposed to involve initial adsorption of dye molecules followed by visible-light-induced photocatalytic transformation on the nanocomposite surface. The suggested degradation pathway is illustrated schematically in Fig. 19, where adsorption enhances dye accessibility to the active sites and promotes subsequent photocatalytic degradation^78,79^.

### Comparative performance analysis

The photocatalytic effectiveness of the AgFeO_2_/PPAC nanocomposite was benchmarked against recently reported studies for the degradation of (NR) and (BBR), as mentioned in Table 7. To maintain scientific relevance, this comparison focuses on literature published within the last decade. A distinctive feature of the AgFeO_2_/PPAC system is its ability to maintain high degradation efficiency at elevated initial dye concentrations, whereas many conventional photocatalysts are optimized for relatively lower concentrations. Specifically, the nanocomposite achieved an efficiency exceeding 99% for both dyes using a minimal catalyst dosage of 5 mg within a 30 min interval. In contrast, several reported systems require higher dosages or extended irradiation times to reach similar benchmarks.

**Table 7:** Comparison of photocatalytic activity of AgFeO_2_/PPAC nanocomposite with other composite based photocatalysts for dye degradation.

Photocatalyst	Dyes	Dye conc. (μg/mL)	Degradation efficiency (%)	Use of magnet	References
rGO (reduced graphene oxide)	NR	25	90.17	No	^69^
Cobalt-mesoporous silica	NR	23	> 81 (220 min)	No	^80^
Gd (Nd-doped)ZIF-8@TiO_2_	NR	20	96.55	No	^81^
TiO_2_ nanorods incorporated with graphene oxide (TiO_2_@GOn)	NR	25	95 (90 min)	No	^82^
AgFeO_2_/PPAC	NR	400	99.8 (30 min)	Yes	This study
ZnO nanoparticles	BBR	28.9	72.8 (90 min)	No	^83^
HAp photocatalyst	BBR	60	80 (120 min)	No	^84^
Co-doped TiO_2_	BBR	30	92.12 (30 min)	No	^85^
Ag nanoparticles	BBR	4–34	90.6	No	^86^
AgFeO_2_/PPAC	BBR	300	99.7 (45 min)	Yes	This study

Beyond its kinetic performance, the nanocomposite offers practical operational advantages. Its inherent magnetic properties facilitate rapid separation from the aqueous phase using an external magnetic field, thereby eliminating the need for energy-intensive filtration or centrifugation. Furthermore, the material demonstrated sustained photocatalytic activity over multiple reuse cycles with marginal efficiency loss. These attributes combined with the use of a biomass-derived support suggest that AgFeO_2_/PPAC is a stable and efficient candidate for dye remediation. While these laboratory results are promising, they provide a strong technical basis for future investigations into the scalability and economic feasibility of the system for industrial wastewater treatment.

## Conclusion

In this study, a novel delafossite AgFeO_2_‑based nanocomposite supported on pomegranate peel activated carbon (AgFeO_2_/PPAC) was successfully synthesized via a hydrothermal route. Structural and surface characterization (XRD, SEM‑EDX, BET, TGA, and FTIR) proved the formation of a phase‑pure delafossite structure free from metallic silver or iron oxide impurities, with AgFeO_2_ uniformly deposited onto a high‑surface‑area porous carbon matrix. The prepared AgFeO_2_/PPAC nanocomposite was evaluated for the adsorptive photocatalytic degradation of anionic BBR and cationic NR dyes from aqueous solutions under visible light irradiation. The material exhibited high removal efficiency toward both dyes in single and binary systems. The experimental data fitted well with the Langmuir isotherm model, suggesting monolayer adsorption, with maximum removal capacities reaching 798.6 mg g^−1^ for NR and 598 mg g^−1^ for BBR. Kinetic analysis showed good agreement with the pseudo‑second‑order model (PSO), indicating the importance of surface interactions. Thermodynamic parameters confirmed a spontaneous and endothermic process. The overall removal performance is attributed to the combined contribution of adsorption and photocatalytic activity. Moreover, the nanocomposite demonstrated good stability and reusability over multiple degradation cycles and its magnetic property enabled easy separation using an external magnet. These findings indicate that the AgFeO_2_/PPAC nanocomposite is a promising and robust platform for the treatment of dye–containing wastewater under visible light.

**Fig. 20:**
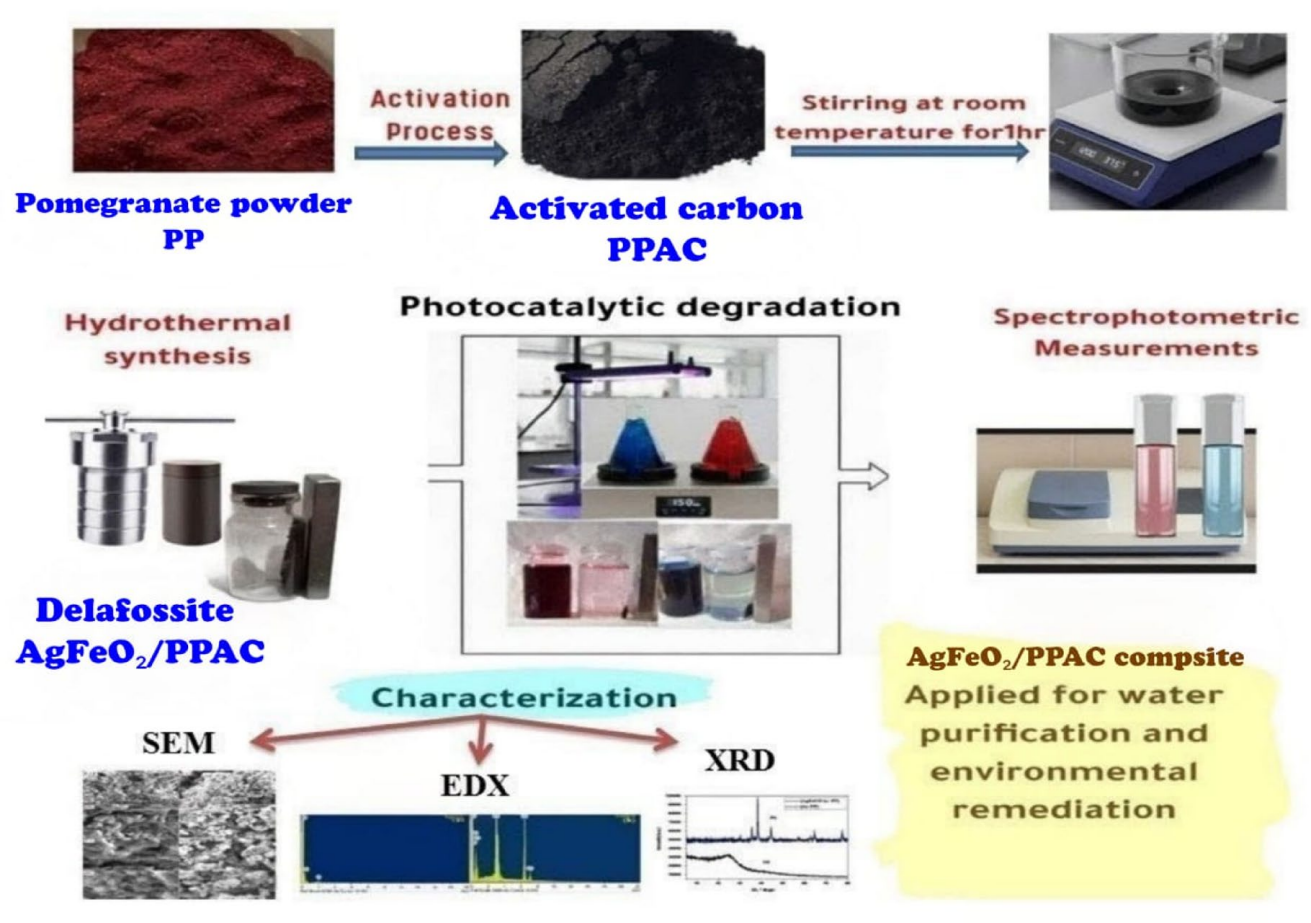
Schematic representation of synthesis, characterization, and application of AgFeO_2_/PPA nanocomposite on the adsorptive photocatalytic degradation of NR and BBR dyes.

Figure 20 represents the synthesis steps of AgFeO_2_/PPAC nanocomposite, including activation of pomegranate powder (PP) into activated carbon (PPAC), hydrothermal synthesis of delafossite AgFeO_2_, material characterization (XRD, SEM, EDX), and final application in photocatalytic degradation for the removal of NR and BBR dyes.

## Supplementary Information

Below is the link to the electronic supplementary material.

Supplementary Material 1

## Data Availability

All data supporting the findings of this study are available within the paper and its Supplementary Information.
