# Supplementary material for: Novel delafossite structured visible-light sensitive AgFeO2/PPAC nanocomposite for efficient adsorptive photocatalytic degradation of cationic and anionic dyes
Source: Sci Rep. 2026 Jul 9;16:21420. doi: 10.1038/s41598-026-60824-z (PMC13350740; doi:10.1038/s41598-026-60824-z)
Supplement: Supplementary file 1 — Supplementary Material 1 [file 41598_2026_60824_MOESM1_ESM.docx]

**Supplementary file**

**Novel Delafossite Structured Visible-Light Sensitive AgFeO_2_/PPAC nanocomposite for efficient adsorpitive photocatalytic degradation of cationic and anionic dyes**

Nora A El-Mahdy^1^, Sayed RH El-Gharkawy^1^ , Asmaa A Serage and Magda A Akl*^1^

^1^Chemistry Department, Faculty of Science, Mansoura University, Mansoura 35516, Egypt

*Correspondence Prof. Magda A Akl. e-mail. magdaakl@yahoo.com

**Table S1.** The classifications, chemical structures and absorption wavelengths of the used organic dyes .

| Name of dye | Color | Chemical structure | Chemical  Formula | Nature | Absorption λmax (nm) |
| --- | --- | --- | --- | --- | --- |
| NEUTRAL RED  ;Toluylene Red  ;3-Amino-7-dimethylamino-2-methylphenazine hydrochloride  ;C.I. Basic Red 5 | 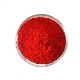 |  | C_15_H_17_ClN_4._HCL | cationic | 530nm  below pH 7.0  460  above pH 8.0 |
| Brilliant Blue R  ;COOMASSIE BRILLIANT BLUE R  ;C.I. Acid Blue 83 | 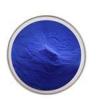 |  | C_45_H_44_N_3_NaO_7_S_2_ | Anionic | 660nm |

| **** | **** |
| --- | --- |

| **** | **** |
| --- | --- |

**Fig.S1.** The BET analysis of PPAC and AgFeO_2_/PPAC bio-composite.

**Fig.S2**. pH_Pzc_ of AgFeO_2_/PPAC composite

**Fig. S3.**The effect of photocatalyst dose on the degradation of NR, and BBR dyes.

**Fig.S4.** The effect of Temperature on the degradation-biosorption of (a) NR, (b) BBR by (AgFeO_2_/PPAC).


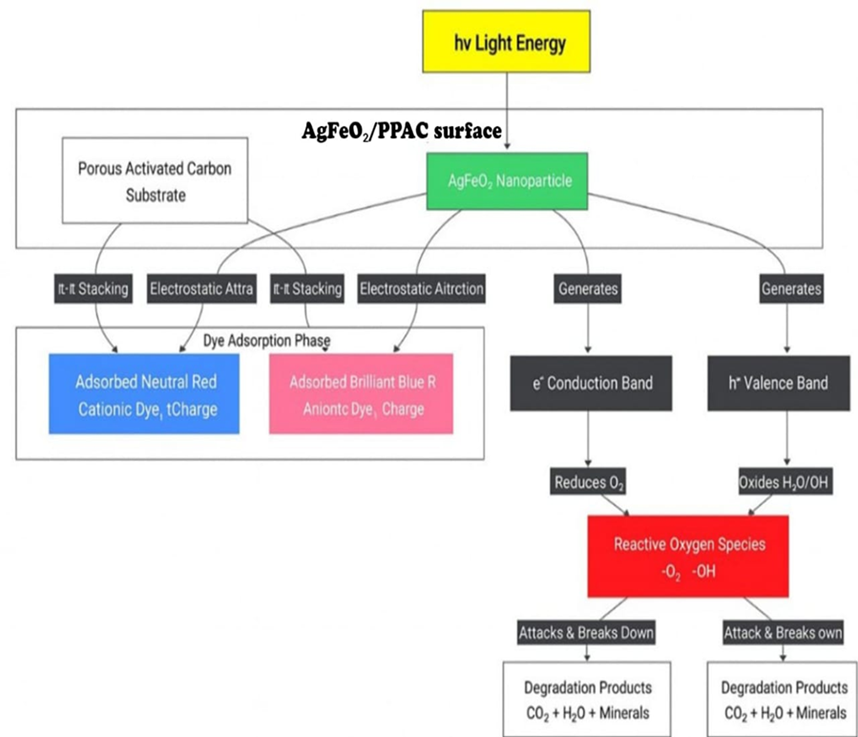


**Fig.S5.** Adsorption–photocatalytic degradation mechanism of dyes over AgFeO₂/PPAC nanocomposite
